# Unlocking male sterility in horticultural crops through gene editing technology for precision breeding applications: presentation of a case study in tomato

**DOI:** 10.3389/fpls.2025.1549136

**Published:** 2025-03-06

**Authors:** Silvia Farinati, Adriana Fernanda Soria Garcia, Samela Draga, Alessandro Vannozzi, Fabio Palumbo, Francesco Scariolo, Giovanni Gabelli, Gianni Barcaccia

**Affiliations:** Department of Agronomy, Food, Natural Resources, Animals and Environment (DAFNAE), Campus of Agripolis, University of Padova, Viale dell’Università, Legnaro, Italy

**Keywords:** plant breeding, male sterility, tomato, F1 hybrids, CRISPR/Cas9, protoplasts, RNP, MYB80

## Abstract

Plant male sterility (MS) refers to the failure of the production of functional anthers, viable pollen grains and/or fertile sperm cells. This feature has great potential in horticultural crops for the exploitation of heterosis through the development of F1 hybrid varieties. MS in plants can occur spontaneously or can be induced artificially by exploiting biotechnological tools, such as the editing of genes involved in spore formation or pollen development. The success of such an approach strongly depends both on preliminary knowledge of the involved genes and on effective procedures for *in vitro* transfection/regeneration of whole plants. Furthermore, according to previous studies based on CRISPR/Cas9 technology, the efficacy of targeting and the resulting mutation profile are critically influenced by intrinsic factors, such as the CRISPR target primary sequence sites and chromatin signatures, which are often associated with varying levels of chromatin accessibility across different genomic regions. This relationship underscores the complexity of CRISPR-based genome editing and highlights the need to identify a precise suitable target. Our paper reports the results obtained for site-specific *in vivo* mutagenesis via a CRISPR/Cas9-mediated strategy applied to the *MYB80* gene, which is a promising target for implementing male sterility in horticultural crops. We highlight the main steps that play a key role in the whole experimental pipeline, which aims at the generation of CRISPR/Cas-edited DNA-free tomato plants. This goal was achieved via protoplast-based technology and by directly delivering a ribonucleoprotein complex consisting of the Cas9 protein and *in vitro* synthesized single guide RNAs that can target different positions of the gene under investigation. Overall findings and insights are presented and critically discussed.

## 1 Introduction

Plant reproduction represents one of the most highly coordinated and fascinating biological processes to be investigated in higher plants, where one of the most remarkable features of this process is the near autonomy of its individual components, permitting the interconversion of numerous reproduction strategies. The success of these strategies is crucial for agricultural productivity, and for this reason, extensive research is continually carried out aimed at a comprehensive understanding and deeper exploitation of these biological processes (Mackenzie, 2012). Among the greatest advances in this field are the induction of male sterility (MS), widely used to exploit heterosis in F1 hybrid seed production in several economically important horticultural crop species (e.g., tomato, eggplant, pepper, onion, cauliflower, and radish) (Rout et al., 2021). Furthermore, the employment of MS can have significant commercial implications, such as the constitution of elite varieties with a greater uniformity, contributing also to food security (Schnable and Springer, 2013; Wei et al., 2021). In plants, MS is defined as the inability to produce, or release, viable pollen grains due to the absence or improper development of anthers, microspores, or male gametes (Kaul, 2012). In MS mutants, abnormalities during cell division within the tapetum have been demonstrated, thereby promoting aborted microgametogenesis engaging genes such as *eme1*/*exs*, *tpd1*, *ams* and *ms1* (Canales et al., 2002; Zhao et al., 2002; Yang et al., 2003). This specific gene pathway has been widely investigated and characterized in model Arabidopsis (Wilson and Zhang, 2009), defining the pollen development transcription factor cascade DYT1-TDF1-AMS-bHLH10/89/91-MYB103/80 (Zhang et al., 2006, 2007; Zhu et al., 2008; Phan et al., 2011; Zhu et al., 2015). A similar gene network regulating pollen and tapetum development was also characterized in rice, including the homologues UDT1-TDR1-TIP2-EAT1, suggesting a high level of conservation in higher plants (Li et al., 2018).

MS can occur spontaneously or be induced by taking advantage of a new generation of biotechnological tools: these biotech-based approaches, focused mainly on molecular biology, genome sequencing and genetic engineering, have contributed to the implementation of new traits in plants, including horticultural crops. Among these, the new breeding techniques (NBTs), also known as precision breeding approaches, represent a quick and effective strategy for the optimization of plant breeding programs (Osakabe et al., 2018; Farinati et al., 2023). CRISPR/Cas9 (*Clustered Regularly Interspaced Short Palindromic Repeats/Cas9*)-based editing technology stands out as one of the most efficient and versatile genetic engineering molecular tools available among NBTs, acting like an accelerator of the process, facilitating precise and efficient targeting (i.e., mutagenesis) of specific sequences, and ensuring the preservation of elite variety genotypes (Zhang et al., 2019; Santillan Martinez et al., 2020). Numerous successful applications in which new traits have been introduced via CRISPR/Cas9 have been reported from the models Arabidopsis (Upadhyay et al., 2013; Yan et al., 2015) and *Nicotiana benthamiana* (Nekrasov et al., 2013; Upadhyay et al., 2013) to many cereals, such as wheat (Upadhyay et al., 2013; Zhang et al., 2016), rice (Jiang et al., 2013; Zhou et al., 2014), maize (Liang et al., 2014; Char et al., 2017), and horticultural crops, such as potato (Wang et al., 2015; Andersson et al., 2017) and tomato (Brooks et al., 2014; Ito et al., 2015). The main complexity of a CRISPR-based genome editing (GE) is the necessity to identify a precise suitable target: according to previous studies, the efficacy of editing process is critically influenced by both target primary nucleotide sequence and epigenetic signatures, as for example the varying chromatin states which could influence the accessibility to the genome across different genomic sites. As evidence of this, a number of molecular techniques have been implemented to predict, when possible, tefficiency and mutation consequences (Allen et al., 2018; Concordet and Haeussler, 2018; Lazzarotto et al., 2020; Xiang et al., 2021), as demonstrated primarily on data from human cells, yeast (*Saccharomyces cerevisiae*), zebrafish (*Danio rerio*), mouse (*Mus musculus*), and rice (*Oryza sativa*) (Wu et al., 2014; Daer et al., 2017; Yarrington et al., 2018; Weiss et al., 2022).

Among the different CRISPR/Cas-based technologies described in the literature, the DNA-free approach, introduced for the first time by Woo and collaborators (Woo et al., 2015), is becoming increasingly promising for its potential increased public acceptance compared with traditional genome editing methods (Entine et al., 2021). In particular, the direct delivery, via protoplast cell cultures, of preassembled ribonucleoprotein (RNP) complexes which are composed of purified Cas9 protein and guide RNA (gRNA), represents one of the most attractive strategies resulting in highly efficient target modifications, avoiding the integration of foreign DNA into plant cells, and eluding the subsequent time-consuming crossings. Moreover, the RNP complex can be rapidly degraded owing to the natural cellular mechanisms of protein and RNA metabolic processing (Chen et al., 2019). This type of cell cultures offers an ideal biological matrix for genetic transformation due to their permeability to exogenous molecules, and ability to guarantee genetic uniformity after *de novo* organogenesis from a single cell, providing a strategic opportunity for producing edited plants and opening new perspectives for breeding purposes (Kim and Kim, 2016; Scintilla et al., 2022). However, to isolate high-quality and viable protoplasts, the subsequent establishment of suspension cells, their transfection efficiency and delivery method (e.g., polyethylene glycol (PEG)) represent some of the key methodological steps responsible for the success of the whole operative pipeline (Demirer and Landry, 2017). Additionally, establishing a successful protocol for inducing the plant regeneration starting from protoplasts culture remains a challenge and a major bottleneck in regeneration systems across many plant species, with different results depending from single variety intra species (Fossi et al., 2019).

Tomato (*Solanum lycopersicum* L.) is among the most important economic crops worldwide. It is an autogamous diploid species with a genome (950 Mb) distributed on 12 chromosomes and is considered a model plant of the Solanaceae family. Since the successful completion of the tomato genome project (TomatoGenomeConsortium, 2012), great progress has been made in the development of improved varieties for different traits, either by traditional or modern breeding strategies, owing to significant progress in the functional analyses of genes involved in various physiological, biochemical, and molecular mechanisms of metabolic pathways (Sun et al., 2006). Since most commercial tomato varieties are F1 hybrids, in genomics era much progress has been made in understanding the pathways of male sterility in tomato plants (Perez-Prat and van Lookeren Campagne, 2002). Although more than 50 male sterile natural mutants have been identified (Gorman and McCormick, 1997), current studies on the induction of male sterility in tomato through the NBT methods are providing successful results (Larriba et al., 2024). Several approaches have allowed breeders to determine the candidate genes, or genetic loci, as potential targets since governing traits of interest (Priyadarsini et al., 2025). Positive results were obtained for the two homologous genes involved in the pollen development transcription factor cascade in tomato. The editing of the first *Solyc02g079810* gene, encoding a basic helix-loop-helix TF (bHLH) and homologue to *AtDYT1* and *OsUDT1*, has confirmed that it is a possible good target candidate for male sterility induction since its knockout mediated by the CRISPR/Cas9 system confers a male sterility phenotype (Jung et al., 2020). The second, *Solyc01g081100*, which is homologous to the *AtbHLH10* and *OsEAT1* genes and potential candidate for the male sterile 32 (*ms32*) mutant (a locus affecting tapetum and pollen development), resulted a good target for a gene editing approach (Liu et al., 2019). Additionally, an aberrant pollen formation was recently induced by CRISPR/Cas9-based knockout of the *SlAMS* gene, which encodes another basic helix-loop-helix (bHLH) TF (Bao et al., 2022).

As previously mentioned, the pollen formation cascade network is composed of members belonging to different gene families whose functional relationships are evolutionarily conserved, with orthologues well characterized in Arabidopsis, rice and tomato (Parish and Li, 2010; Xu et al., 2014). Among these genes, the R2R3 transcription factor MYB80 plays an important role in different stages of anther development in several species (Phan et al., 2012), with homologues identified in canola, wheat, rice and cotton characterized by highly conserved R2R3 DNA-binding domains, although the C-terminal domain is highly variable (Phan et al., 2011). In *A. thaliana*, for example, a mutation in the first exon of *MYB80* (also referred to as *MYB103*) is responsible for a sterile male phenotype, where tapetum development and callose dissolution are altered in defective plants (Zhang et al., 2007). Starting from these considerations, we present the first insights and preliminary evidence for evaluating the potential of targeting the orthologous *MYB80* gene (*SlMYB80–Solyc10g005760*) through a DNA-free CRISPR/Cas9 approach based on protoplast-mediated delivery of a preassembled RNP complex via transient transfection. Given the complexity of the whole experimental procedure, which includes several critical points, in this manuscript we focused on and critically discussed only the key points to evaluate how much they could influence the success of the entire process, in order to apply this strategy in the future with the purpose of generating GE-deriving male sterility tomato plants.

## 2 Materials and methods

### 2.1 Plant material and growth conditions

Tomato seeds (*Solanum lycopersicum* L.) cv. Microtom were germinated on moist filter paper at 28°C. The seedlings were then transferred to pots and grown in the greenhouse of the experimental farm ‘Lucio Toniolo’ - University of Padova - Italy (GPS coordinates: 45°21′ N, 11°58′ E, 6 m a.s.l.). For subsequent molecular investigations, flower buds were staged according to Brukhin’s (Brukhin et al., 2003) classification based on phenological flowering development, and the results are summarized in **Table 1**. Anthers were carefully isolated from buds collected at different stages, frozen with liquid nitrogen and stored at -80°C.

**Table 1:** Tomato floral growth stages corresponding to physiological events described by (Brukhin et al., 2003).

Length of buds (mm)	Phenological Stage (S)	Physiological event
2	8	Deposition of callose around the Pollen Mother Cell (PMC)
**3**	**9**	Meiosis initiation
**3,5**	**10**	Release of tetrads
**4**	**11**	Resorption of callose
**5**	**12**	Release of the tetrads
6	13	Degeneration of tapetum - Starting phase
**7**	**15**	Degeneration of tapetum - Completed
8	16	Pollen mitosis
10	20	Anther dehiscence

The bold stage numbers indicate the samples used for subsequent RNA extraction and expression analyses.

For *in vitro* culture, tomato Microtom seeds were surface sterilized for 5 min by washing with 50% bleach solution, to which a pair of drops of Tween-20 were added, followed by at least three washes with sterile water. The seeds were subsequently placed in hormone-free Murashige & Skoog medium including B5 vitamin mixture (MSM, Duchefa Biochemie; Haarlem; The Netherlands) supplemented with 3.0% sucrose and 0.8% agar and then placed in a growth chamber at 25°C with a 12 h light/dark cycle and an intensity of 35 µmol m^-2^s^-1^. After 4–5 weeks, the *in vitro*-grown plant material was clonally propagated by microcutting, transferred to fresh, hormone-free MSM, and grown for another 4 weeks.

### 2.2 Bioinformatics analysis of tomato candidate gene

The predicted amino acid sequence of SlMYB80 was retrieved from the Solanaceae Genomics Network database (SGN 3.0; http://solgenomics.net) and aligned with the putative corresponding previously studied orthologous genes of *Arabidopsis thaliana* (Zhang et al., 2007), *Gossypium hirsutum* (Xu et al., 2014), *Oryza sativa* (Pan et al., 2020), and *Cichorium intybus* (Palumbo et al., 2019). The sequences under investigation for *Arabidopsis thaliana* (AtMYB80 - AT5G56110), *Oryza sativa* (OsMYB80 - LOC_Os04g39470), *Gossypium hirsutum* (GhMYB80 - LOC_107931145*)* and *Cichorium intybus* (CiMYB80 - GenBank: MK285054.1) species were retrieved from Phytozome (http://www.phytozome.net) and BLAST (https://blast.ncbi.nlm.nih.gov/) with default settings using the full-length amino acid sequence of Arabidopsis MYB80 as a query. Amino acid alignments were performed with ClustalW in MEGA 7.0 setting default parameters.

The exon/intron gene structure of *SlMYB80* was predicted with the Gene Structure Display Server (GSDS; http://gsds.cbi.pku.edu.cn/). Gene structure prediction was then confirmed in cv. Microtom via Sanger sequencing and alignment of nucleotide sequences corresponding to the full-length genomic coding sequence and full-length transcript. Furthermore, a nucleotide sequence corresponding to the putative basal promoter region of the *SlMYB80* gene was amplified and cloned. The prediction of the TATA box and transcription start site (TSS) was performed via Softberry-TSSP software (http://www.softberry.com).

### 2.3 Nucleic acid extraction

Genomic DNA (gDNA) was extracted from young leaves of tomato plants via a Qiagen DNA Extraction Kit (Qiagen, Haan, Germany) following the manufacturer’s instructions. Leaf tissue was fragmented via TissueLyser II (Qiagen, Haan, Germany). DNA concentrations were measured with a Nanodrop spectrophotometer (NanoDrop Technologies, Wilmington, USA), and the DNA was diluted to a final concentration of 30 ng/μl in TE buffer (pH 7.0) for further experiments.

Total RNA was extracted from leaves and anthers via a RNeasy Plant Kit (Qiagen, Haan, Germany) following the manufacturer’s instructions. The genomic DNA was then removed via DNase digestion (Life Technologies, Catalogue # 18068–015) according to the manufacturer’s instructions.

### 2.4 Primer design and PCR assays

All primers used in this work were designed via the Primer-BLAST online tool and subsequently verified with the IDT oligoanalizer tool (OligoAnalyzer Tool - Primer analysis and Tm Calculator | IDT). All primers were synthesized by Invitrogen (Thermo Fisher, USA). The nomenclature and sequences of all the oligonucleotides are listed in **Supplementary Materials**, **Supplementary Table S1**.

PCR assays were performed using 1× Platinum Multiplex PCR Master Mix with the addition of a GC enhancer (Applied Biosystems, Carlsbad, USA), 0.1 μM for each specific primer (forward and reverse), and sterile water to volume. A 9600 Thermal Cycler (Applied Biosystems) was used, and the conventional experimental conditions were set on the basis of the primer sequences employed and the length of the fragment used for amplification.

### 2.5 Cloning and sequence analysis

The PCR-amplified fragments were subsequently cloned and inserted into the pCR2.1-TOPO vector (Thermo Fisher, USA) following the manufacturer’s instructions. The plasmids were then isolated and purified with the QIAprep Spin Miniprep (Qiagen, USA). The nucleotide sequence of each insert was verified mediating Sanger sequencing using the universal M13 forwards and reverse primers.

### 2.6 cDNA synthesis and qPCR analysis

Anther tissues were collected from buds of *in vivo*-grown tomato plants for subsequent expression analyses. Each tissue sample comprised three biological replicates, each consisting of a pool of six buds from at least three plants. cDNA was synthesized from 1 µg of total RNA via the PrimeScript™ RT Reagent Kit following the manufacturer’s protocol (Takara, Dalian, China). The qPCRs were performed in a Thermo Fisher QuantStudio 3 real-time PCR instrument (Thermo Fisher) via 96-well optical PCR plates (Applied Biosystems, Foster City, USA) with SYBR^®^ Green Real-time PCR Master Mix as the detection system. Output data analyses and normalization were performed with QuantStudio Design and Analysis Software v1.4 (Thermo Fisher). The data were quantified with the 2^−ΔΔCt^ method based on Ct values of the housekeeping gene glyceraldehyde-3-phosphate dehydrogenase (*GAPDH*) (Lovdal and Lillo, 2009). All primers used for setting qPCRs are listed in the **Supplementary Materials**, **Supplementary Table S1**.

### 2.7 *In situ* hybridization

Tomato buds were sampled from *in vivo*-grown plants at two different stages, corresponding to the S10 and S15 phenological growth stages. The buds were fixed in 0.1 M phosphate buffer supplemented with 4% paraformaldehyde. The samples were subsequently dehydrated in a series of ethanol/xylene and embedded in paraffin. After that, the samples were sliced with a Leica microtome into longitudinal Sections 10 μm thick and placed on poly-L-lysine slides. Several progressive ethanol/xylene series were used to remove the paraffin.

An amplified *SlMYB80*-specific fragment of 200 bp from cDNA generated with the primers III_ex_01_Fw/III_ex_02_Rev (**Supplementary Table S1**) was cloned and inserted into a TA cloning vector, and the recombinant plasmid was used for transforming TOP10 *E. coli*-competent cells. The insert was amplified from the plasmid with M13 forward and reverse primers, used as the template for sense (SP6) and antisense (T7) RNA probe synthesis and labelling via the Roche DIG RNA labelling kit (SP6/T7) (Roche, Basel, Switzerland). Hybridization was performed for 12 h at 42°C in a buffer containing 10 mM Tris–HCl (pH 7.5), 300 mM NaCl, 50% deionized formamide, 1 mM EDTA (pH 8), 1 × Denhardt’s solution, 10% dextran sulfate, 600 ng/ml total RNA and 60 ng of the corresponding probe. Detection was performed following the DIG detection kit instructions (Roche) using anti-DIG AP and NBT/BCIP as substrates. A digital camera attached to a Nikon eclipse Ts2R microscope was used to observe the brightfield images of the sections. As a dependable outcome, only hybridization signals exhibiting a regular pattern were reported.

### 2.8 Guide RNAs design and *in vitro* screening

Guide RNAs (gRNAs) design were performed using the CRISPOR digital platform (https://crispor.tefor.net/). The best gRNAs were selected according to scores that evaluate potential off-targets in the tomato genome and predict on-target activity. The potential gRNAs for subsequent editing were chosen to target the promoter region and the first and third exons of the *SlMYB80* gene locus. To synthesize and test the efficiency of the specific nucleotide sequence of the gRNAs predicted and selected via bioinformatics tools, the Guide-it Complete sgRNA Kit (Takara, Cat. No. 632636) was used following the manufacturer’s instructions.

### 2.9 Isolation of tomato protoplasts and determination of yield and viability

The protoplasts were isolated from leaf tissues collected from *in vitro* grown young plants of 3 to 4 weeks after microcutting propagation. The isolation and purification of tomato protoplasts were performed according to the protocol of Yoo et al. (2007) and Bertini et al. (2019) for the isolation of protoplasts from lettuce leaves and grapevine calli, respectively. The final adapted protocol for the tomato system also followed the guidelines of Nicolia et al. (2021) and Tan et al (Tan et al., 1987b). All the solutions used and the procedure details for protoplast isolation are available in the **Supplementary Materials**, **Protocol S1**.

The protoplast yield was determined via a haemocytometer under a Nikon Ts2R microscope (NIKON Europe, Amsterdam) as follows:


$$\begin{array}{c} \text{Protoplast yield }\left(\text{pieces}/\text{g FW}\right)\\ \;=\;[\text{number of protoplasts }\left(\text{number of protoplasts}/\text{ml}\right)\\ \;\times \text{ protoplast volume }\left(\text{ml}\right)]/\text{total leaf mass }\left(\text{gFW}\right) \end{array}$$

The viability was observed under a fluorescence microscope and estimated with one microlitre of a solution of 0.01% (w/v) fluorescein diacetate acetone (FDA) added to 100 µl of protoplast suspension and incubated in the dark for 10 min via the following mathematical equation:


$$\begin{array}{c} \text{Viability}\;\left(\%\right)\;=\;(\text{number}\;\text{of}\;\text{FDA}\\ -\text{positive}\;\text{protoplasts}/\text{total}\;\text{number}\;\text{of}\;\text{protoplasts}\;\text{counted}\;)\;\times \;100 \end{array}$$

The protoplasts were then diluted in MMG solution for subsequent transfection events. At least three biological replicates and three technical replicates were used to optimize the protocol.

### 2.10 Transfection of tomato protoplasts

The transfection procedure was performed with a Cas9-GFP fusion protein (Sigma−Aldrich). The lyophilized protein was suspended in the supplied reconstitution solution to achieve a concentration of 5 mg/ml. Following the manufacturer’s instructions, the components were incubated for 10 min at room temperature in the dark before protoplast transfection. The PEG-mediated protoplast transfection method was adapted from or based on the methods described by Yoo et al. (2007) and Yuan et al (Yuan et al., 2023). In detail, freshly isolated protoplasts were kept at 4°C for a maximum of 1 h to stabilize the just released protoplasts. Then, 2 × 10^5^ protoplasts were suspended in 200 μl of MMG buffer, gently mixed with 15 μg of Cas9-GFP fusion, and mixed with 220 μl of 40% (w/v) PEG 4000 solution prepared in 0.2 M mannitol and 0.1 M CaCl_2_. All the solutions used and the procedure details for protoplast transfection are available in the Protocol S1. The resulting mixture was incubated at RT in the dark for 15 minutes. After centrifugation for 5 minutes at 100×g at RT, the supernatant was removed, and the protoplasts were washed twice under the same conditions. Finally, the pellet was suspended in 1 ml of W1 solution and incubated in the dark at room temperature. After 15 min, the fluorescence signal of the GFP was visible with a Nikon Ts2R microscope (NIKON Europe, Amsterdam) (excitation wavelength of 488 nm and emission wavelength of 503-530 nm). The transfection efficiency was estimated based on the number of GFP-transformed cells.

For RNP complex assembly targeting different regions of the candidate gene under investigation, gRNAs were synthesized via the GeneArt Precision gRNA Synthesis Kit (Invitrogen, Thermo Fisher Scientific, Waltham, MA, USA) according to the manufacturer’s instructions. After several ratios of Cas9 (15 μg) to gRNA (i.e., 2:1, 1:1, and 1:2) were tested, a 1:2 ratio (w/w) was identified as optimal for the experiments in this study.

Untransfected protoplasts treated with PEG only were used as negative control samples for transfection (NTCs). For the transfection process, two hundred microlitres of the protoplast suspension (2*10^5^ protoplasts) were mixed with 20 microlitres of the RNP complexes previously assembled, followed by the addition of 220 µl of 40% (w/v) PEG solution and incubation for 15 min at RT. After centrifugation for 5 minutes at 100×g at RT, the supernatant was removed, the pellet was washed twice with W5 solution and finally resuspended in 1 ml of W1 solution, followed by 48 hours of incubation in the dark, followed by genomic DNA extraction.

A quantitative assessment of the genome editing was carried out with the online software TIDE (Tracking of Indels by Decomposition, https://tide.deskgen.com) (Eva et al., 2014; Brinkman and van Steensel, 2019). The program calculates the range and frequency of tiny insertions and deletions (INDELs) produced in a cell pool after the application of genome editing technologies (e.g., CRISPR/Cas9), determining the most common forms of INDELs. Following TIDE instructions for the samples preparation the DNA was extracted from the transfected protoplasts and from the control group via a Qiagen DNA Extraction Kit (Qiagen, Haan, Germany) and used for a PCR amplification via primers designed on gRNA recognition site flanking regions. After purification, the PCR-purified products were sequenced via the Sanger method. Following TIDE guidelines, the chromatogram sequence files obtained from Sanger sequencing of the transfected and control groups, without any manipulation, were used as inputs, along the guide sequence, with default setting.

### 2.11 Chromatin extraction and ChIP target analysis

Chromatin extraction/purification and subsequent chromatin immunoprecipitation (ChIP) analysis were performed on a protoplast population isolated from *in vitro* leaf tissue, as described in the previous sections. Considering the technical limits of the procedure, chromatin extraction was performed after pooling three biological replicates corresponding to three independent protoplast extractions, starting from at least 5*10^6^ protoplasts each. After recovering the protoplast cells via centrifugation at 500xg for 5 minutes at RT, the cell pellet was washed twice with PBS buffer (pH 7.4) and then fixed with 1% formaldehyde for 15 minutes at RT. The following chromatin extraction and immunoprecipitation phases were performed as reported in literature (Canton et al., 2022), adjusting specific technical steps. In particular, the chromatin pellet was suspended in 500 µl of lysis buffer (50 mM HEPES pH 7.6, 150 mM NaCl, 1 mM EDTA, 1% Triton X-100, 0.1% deoxycholate, 0.1% SDS, 10 mM Na-butyrate, and protease inhibitor cocktail–Sigma-). After sonication to obtain 200–300 bp fragments, a fraction was saved, and after reverse cross-linking with 0.2 M NaCl for 16 h at 65°C, it was used for quantification and as input in the following PCR evaluation. For the subsequent immunoprecipitation phase, 5 µg of chromatin was used for each antibody reaction. The histone−DNA complexes were immunoprecipitated via Dynabeads protein G (Invitrogen), and the appropriate antibodies against the following proteins were added: α-H3K9ac (Millipore, Cat. 07-352), α-H3K4me3 (Active Motif, Cat. 39159), and α-H3K27me3 (Millipore, Cat. 07-449), followed by incubation overnight at 4°C. Finally, the DNA was recovered in TE buffer, treated with RNAse I for 30′ at 37°C and proteinase K for 1 h at 42°C, and extracted once with phenol−chloroform and with a QIAquick PCR purification kit (QIAGEN). Two independent immunoprecipitations for each Ab used were performed, and a chromatin aliquot processed like other samples but without the addition of any antibody (No Ab sample) was used as a background control. One microlitre of this ChIPed DNA and an appropriate dilution of input were used for the following qPCR analyses. For each target region under investigation, the sequences of the working primers used in the qPCR are reported in the **Supplementary Table S1** (i.e., Prom_04_Fw/Prom_05_Rev for the basal promoter region, I_ex_01_Fw/II_ex_01_Rev for the first exon, III_ex_01_Fw/III_ex_02_Rev for the third exon). qPCR data analyses were performed and significant differences in the level of each analysed histone mark were assessed via Student’s t tests (Rossi et al., 2007).

## 3 Results

### 3.1 Multiple peptide alignment of SlMYB80 and other potential homologous MYBs

**Figure 1:**
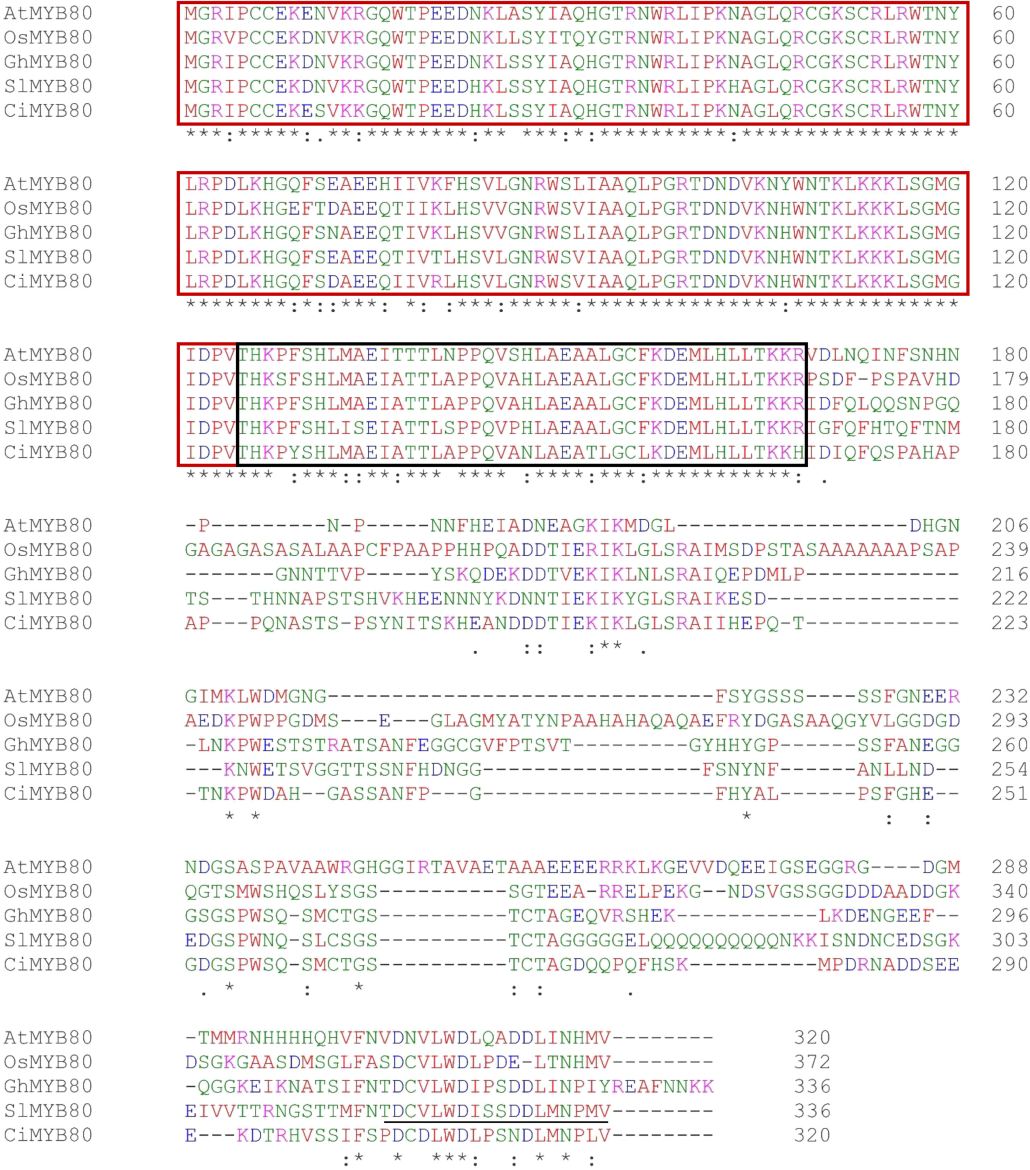
Alignments of MYB80 peptides. Multiple alignment of peptide sequences of five different MYB80 potential orthologues via the ClustalW program. The colours represent the different amino acid properties: red (small and hydrophobic), blue (acidic), purple (basic), and green (hydroxyl, amine). The red box indicates the R2R3 MYB domain. The black box indicates the 44-aa conserved domain. The unhighlighted region corresponds to the C-term region, which includes 18 similar amino acids (underlined). The following abbreviations indicate the relative species: At, *Arabidopsis thaliana*; Os, *Oryza sativa*; Gh, *Gossypium hirsutum*; Sl, *Solanum lycopersicum*; Ci, *Cichorium intybus*.

As a starting point of the investigation, a multiple alignment was performed for elucidating the potential relationships between SlMYB80 and other MYB proteins previously studied and related to the male sterility in other plant families (**Figure 1**). Despite the high phylogenetic distance between monocot and dicot species from which the sequences were recovered, all the MYB80 members ranged from 320 to 370 amino acids in length and showed a degree of similarity from 60 to 70%, depending on the genetic distance between species. The alignment shown in **Figure 1** reveals significant conservation of the putative R2R3 MYB domain in the first 124 amino acids. A region following the MYB domain, consisting of 44 amino acids, was also shown with a single similar pattern among all species, terminating with KKR peptide conservation at position 169. A very variable region of approximately 130 amino acids appears in all the sequences, followed by 18 similar amino acids in the C-term region, which can slightly differ from species to species.

### 3.2 Prediction and validation analyses of the *MYB80* genomic locus in the tomato cultivar Microtom

**Figure 2:**
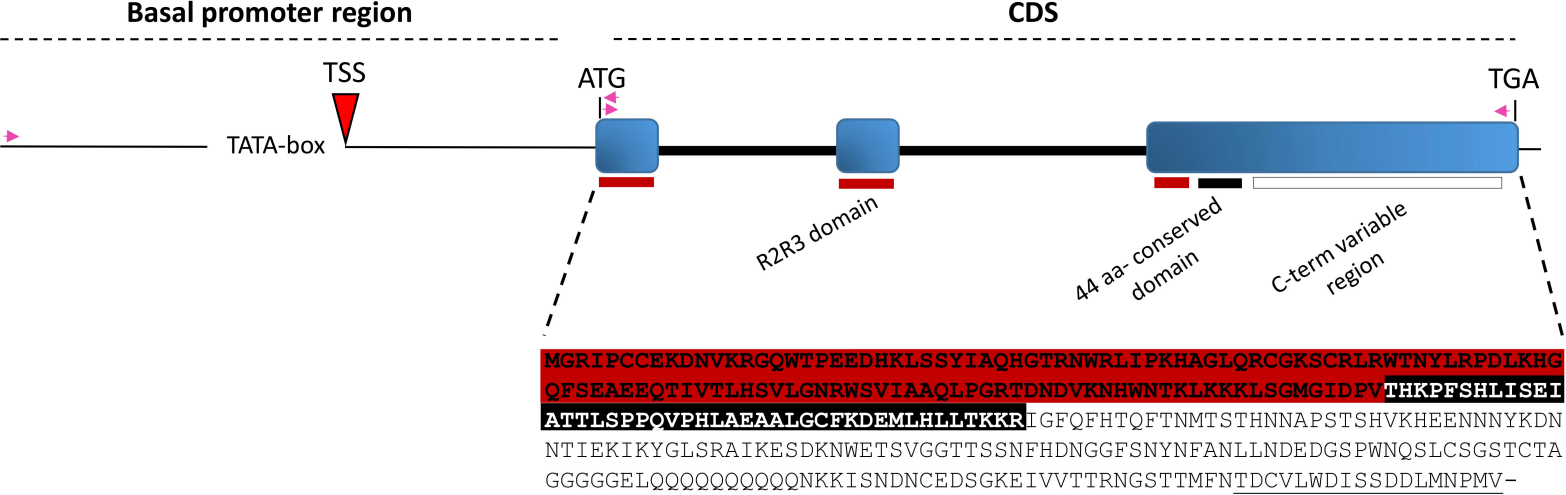
Schematic representation of the SlMYB80 genomic locus, including the upstream basal promoter region and CDS. The relative positions and orientations of the primers employed for locus amplification are indicated with purple arrows. The predicted TATA box and TSS in the promoter region are indicated. The exons are indicated with filled light blue rectangles, whereas the introns are indicated with thick black lines in the CDS region. The red, black and white rectangles map the exons of the nucleotide regions coding for R2R3, the 44-aa conserved and the variable C-term domains, respectively. The 18 similar amino acids in the C-term domain are underlined in the peptide sequence.

To determine the *SlMYB80* allele gene sequence in the tomato cv. Microtom under investigation, the primer pairs I_ex_01_Fw and III_ex_03_Rev (**Supplementary Table S1**) were designed for PCR amplification of the full-length genomic CDS, followed by Sanger sequencing. The sequence results confirmed complete nucleotide sequence identity to the reference genomic sequence retrieved from the SGN database of tomato genome version SL3.0. The graphical representation of the exon/intron organization was obtained via the GSDS online tool, which highlighted a composition of three exons (133, 130 and 748 bp in length) and two introns (370 and 514 bp in length) (**Figure 2**). This exon−intron organization in cv. Microtom was then confirmed via amplification of a cDNA sequence from RNA extracted from anther tissue collected and pooled at different stages, confirming that the full-length CDS transcript could encode a predicted putative 336-aa peptide (**Figure 2**). Additionally, a genomic region corresponding to 1,159 bp upstream of the ATG site was amplified via the primers Prom_01_Fw and I_ex_01_Rev (**Supplementary Table S1**) to map the putative TSS in the *SlMYB80* locus via the Softberry-TSSP software tool. The putative TSS was predicted to be -522 bp upstream of the annotated ATG (**Figure 2**).

### 3.3 Tissue-specific expression analysis of SlMYB80

**Figure 3:**
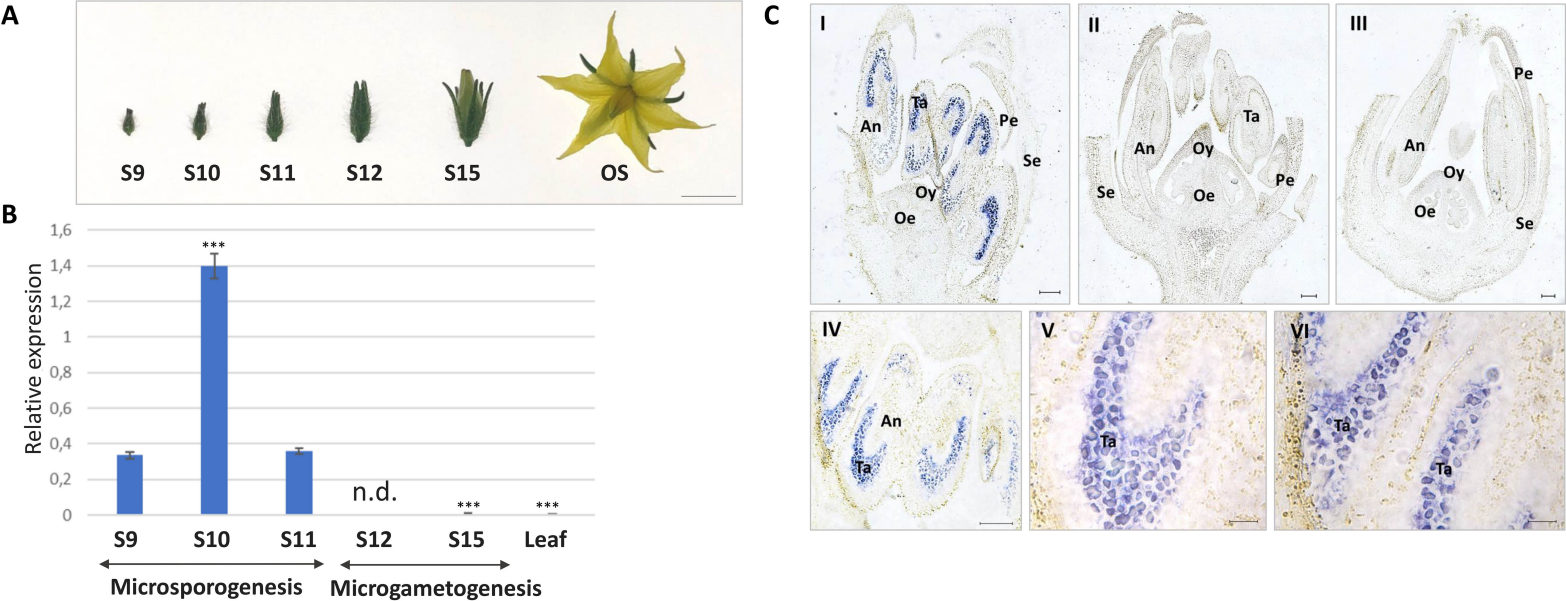
Tissue expression analysis of *SlMYB80* transcripts. **(A)** Morphology of floral buds collected at different phenological stages. S: phenological stage. OS: opened flower stage showing the complete growth. This stage is not considered in following expression analyses. Bar: 5 mm. **(B)** Expression analysis of *SlMYB80* in anthers collected from buds at the S9, S10, S11, S12 and S15 growth stages. The normalized expression level of the *SlMYB80* transcript was measured via real-time qRT−PCR. The data were analysed to determine gene expression via the 2^-△△CT^ method. The error bars represent the standard error. Asterisks indicate statistically significant changes: *** = p≤ 0.001. n.d.: not detected. **(C)** *In situ* hybridization analysis of longitudinal bud sections collected at the S10 (I, II) and S15 (III) growth stages. Hybridization signal on anthers collected from S10 (IV, V) and S11 (VI). Hybridization was carried out with antisense (AS, I, III–VI) and sense (S, II) probes. Se, sepals; Pe, petals; Oy, ovary; Oe, ovules; An, anther; Ta, tapetum. Bars: 100 µm.

The expression level of *SlMYB80* was evaluated in anthers collected from floral buds of *in vivo*-grown plants (**Figure 3A**). The classification of floral buds for subsequent collection was based on Brukhin et al. (2003) classification and is summarized in **Table 1**. Specifically, expression analysis was performed on anthers collected from bud samples corresponding to stages S9, S10, S11, S12 and S15, in which microsporogenesis and microgametogenesis occur. For expression analysis, the pairs of primers III_ex_01_Fw/III_ex_02_Rev and GADPH_Fw/GADPH_Rev were used for the *SlMYB80* and *GADPH* genes, respectively (**Supplementary Table S1**). The analysis revealed the highest level of *SlMYB80* transcripts in samples derived from stages 9, 10 and 11, followed by a dramatic reduction in stages 12 and 15, and in the leaf control samples. These preliminary observations support the hypothesis that *SlMYB80* may be a key candidate for male sterility induction and predominantly functions during microsporogenesis (**Figure 3B**).

The spatial tissue localization of the *SlMYB80* transcripts was investigated through *in situ* hybridization of whole buds (including anthers). The hybridization signal in S10 bud longitudinal sections, derived from an RNA antisense (AS) probe complementary to the *SlMYB80* transcript, revealed that *SlMYB80* mRNA was expressed exclusively in anthers (**Figure 3C**, I, IV), whereas no signal was detected in other floral tissues. At the same S10 growth stage, no hybridization signal was detected in the negative control (hybridized with a RNA sense-S-probe) (**Figure 3C**, II). In sections of more mature floral buds corresponding to the S15 growth stage, the hybridization signal was faint or undetectable (**Figure 3C**, III). The specificity of signals with the antisense (AS) probe is evident at the level of the tapetum and endothecium tissues of anthers collected from both S10 and S11 (**Figure 3C**, IV–VI).

### 3.4 Improving the isolation of protoplasts and transfection conditions

**Figure 4:**
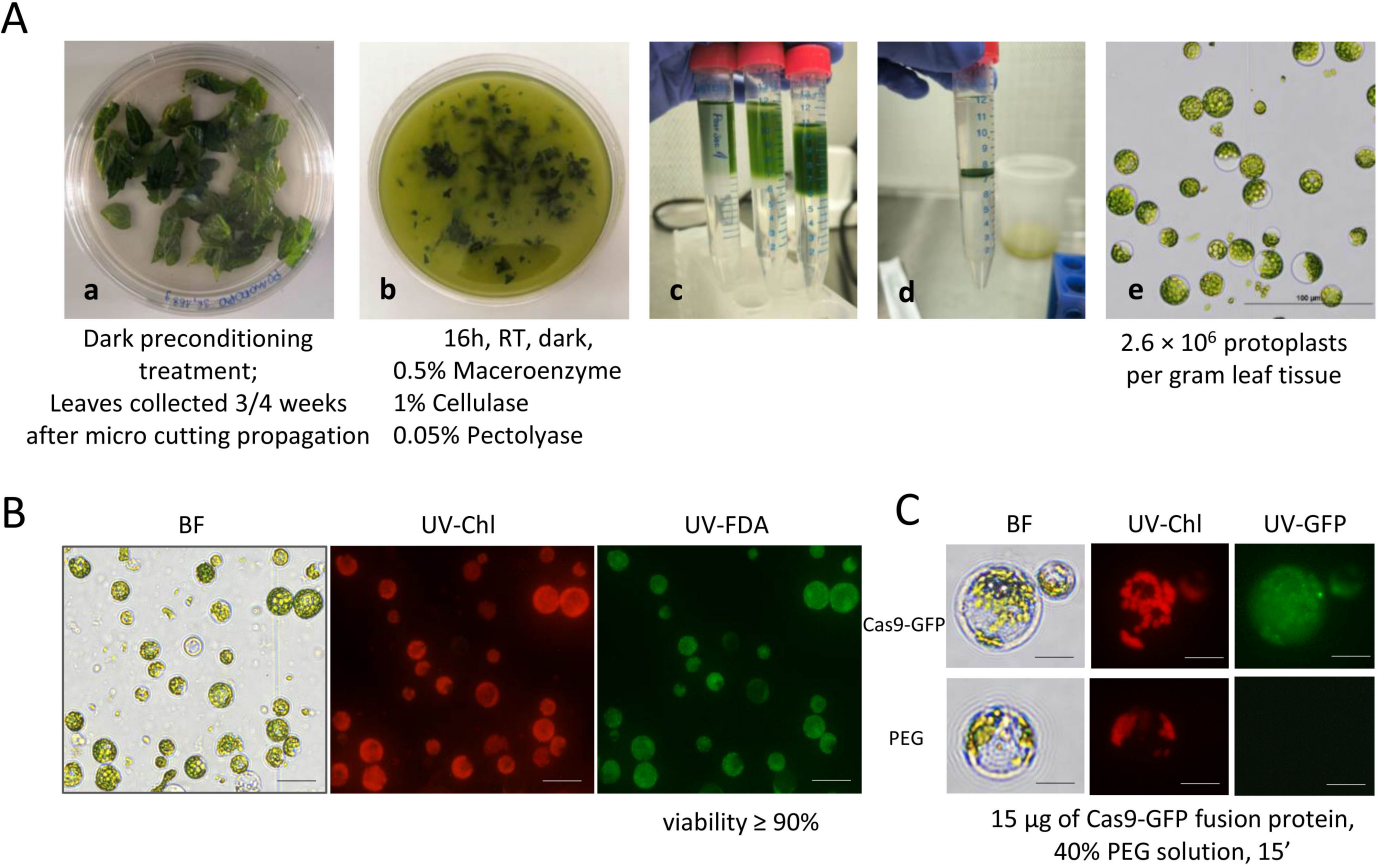
Isolation of protoplasts and overview of the transfection conditions. **(A)** Schematic overview of the stages of the protoplast isolation process from *in vitro* cultivated tomato cv. Microtom. The main steps are summarized as follows: (a) Selected tomato plants from *in vitro* culture were used for protoplast isolation; (b) Sliced leaves were incubated in the enzymatic solution after 16 h; (c) Sucrose gradient purification was performed before centrifugation; (d) Dark green rings containing released intact protoplasts at the interface of sucrose solution; and (e) Freshly isolated green protoplasts under a microscope 40 X. Bar: 100 µm. **(B)** Viability test after FDA-approved treatment. Bars: 50 µm. **(C)** Intracellular localization of Cas9-GFP in tomato protoplasts. Bars: 20 µm.

To apply protoplast-based technology for obtaining DNA-free CRISPR/Cas9 plant material, a specific procedure was optimized for isolating protoplast cells from *in vitro*-grown tomato plants. In particular, improvements in the yield and viability of isolated protoplasts were obtained by adapting previously published methods applied for tomato (Nicolia et al., 2021; Liu et al., 2022), grapevine (Bertini et al., 2019) and model species (Yoo et al., 2007). The main key steps of the protoplast isolation procedure are shown in **Figure 4A**, in which the main experimental conditions employed (e.g., plant cutting age, enzymatic digestion composition, temperature/time, and preconditioning treatment) are also indicated. The implemented procedure allowed us to obtain the best yields, shape and viability, isolating healthy cells from many nonviable cells during the protoplast isolation process. The enzymatic solution formula and the incubation times adopted were demonstrated to be effective: the best results of isolation were obtained from leaves collected from 3 to 4-week-old propagated *in vitro* plants, which were previously preconditioned at 4°C for at least 8 h in the dark, and subjected to 0.5% Maceroenzyme, 1% cellulase or 0.05% pectolyase enzymatic solution (**Figure 4A**, a-b) (for more details, see Materials and Methods section). Moreover, the addition of sucrose and proper gradient formation enabled the removal of broken, nonviable protoplasts from the final mixture (**Figure 4A**, c-d). The isolated protoplasts had a uniform spherical shape, indicating complete cell wall digestion, with diameters ranging from 20–60 µm, and no aggregates of undigested cells were observed. A final average yield of up to 2.6 × 10^6^ protoplasts per gram of leaf material was estimated by counting the cells with a haemocytometer (**Figure 4A**, e). Protoplast viability was tested via FDA staining, which distinguishes viable protoplasts from nonviable protoplasts through fluorescent signal emission. The FDA assay showed that the average viability was greater than 90% (**Figure 4B**).

The development of an efficient and reproducible protocol allowed us to proceed with the following transfection phase. As a first step, a Cas9-GFP fusion protein was utilized, with GFP acting as a visual marker to monitor transfection. In addition, a PEG-mediated transfection method was employed since it is considered a standard procedure to introduce DNA into protoplasts, as demonstrated in several plant species (Yoo et al., 2007; Ohnuma et al., 2008; Shen et al., 2014). In this work, a modified PEG-mediated protoplast transfection protocol was established based on the method described by Yoo et al (Yoo et al., 2007). After several attempts to determine the optimal transfection conditions (in terms of the quantity of the fusion protein, PEG concentration and time of transfection), freshly isolated protoplasts (2*10^5^) were transfected with 15 μg of Cas9-GFP fusion protein supplemented with 40% PEG solution for 15 minutes. Once the incubation period was complete, the GFP signal was monitored under a microscope at fixed time intervals after reaching 24 h (i.e., 15 min, 1–12–24 h), after which the protoplast suspension was kept in the dark at RT. Notably, 15 min post transfection, prominent signal nuclear localization related to Cas9-GFP in protoplasts was observed (**Figure 4C**), with a transfection efficiency ranging from 30–40%. Similar signals and no significant changes were observed at other time points at 1-12-24 hours (data not shown).

### 3.5 Design and *in vitro* validation of gRNAs at the *SlMYB80* locus

**Figure 5:**
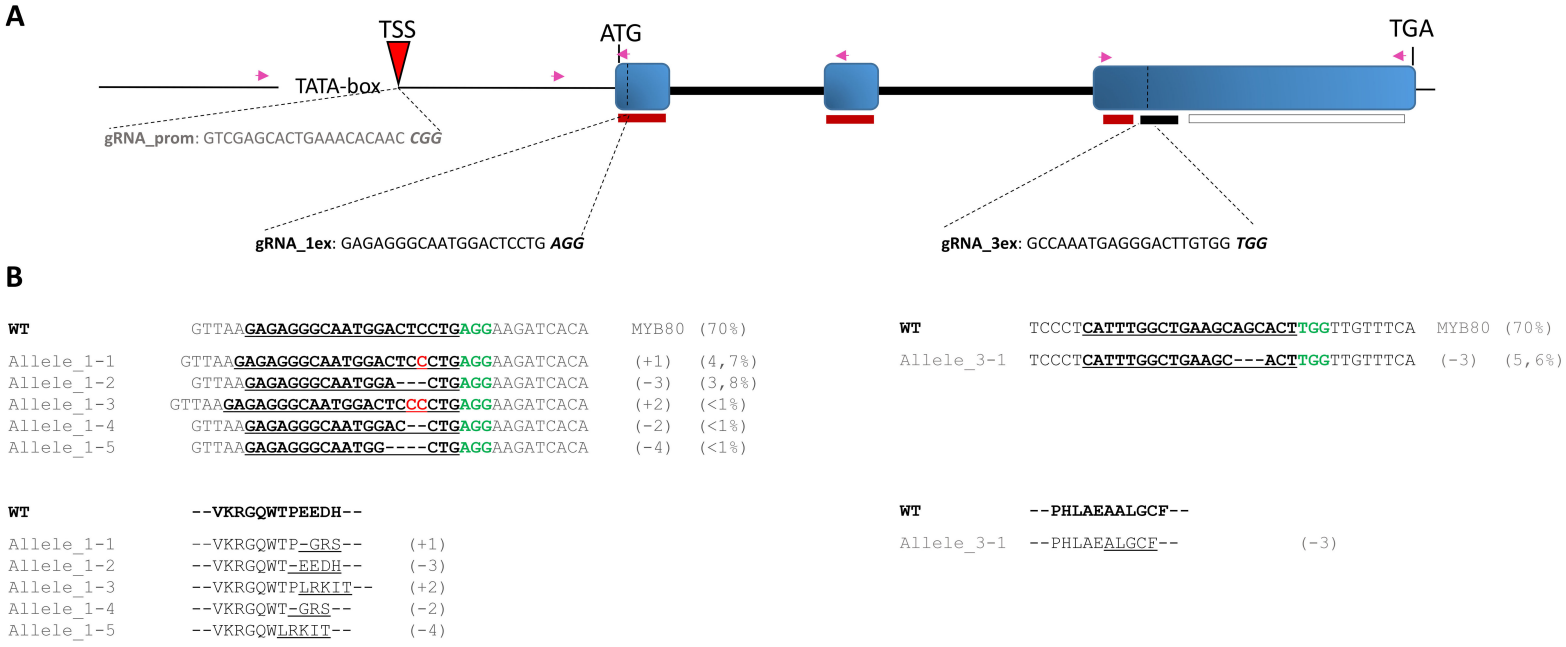
Schematic representation of CRISPR/Cas9-mediated target mutagenesis of the *SlMYB80* gene. **(A)** Schematic representation of the *SlMYB80* genomic locus, including the upstream basal promoter. The position and relative sequences of the three gRNAs (gRNA_prom, gRNA_1ex, and gRNA_3ex) are visualized. The PAM motif sequences are represented in italics and bold. The functional domains in the CDS targeted by gRNA are reported with thin red and black rectangles, corresponding to the R2R3 and 44-aa conserved domains, respectively. The relative positions and orientations of the primers employed for amplification of specific regions are indicated with purple arrows (**Supplementary Table S1**). **(B)** Genotyping by Sanger target sequencing after transfection with RNP1 and RNP3 in R1 replicates. The DNA sequence of each allele, and the predicted *in silico* translation-derived amino acid sequence, was aligned with the wild-type (WT) allele, highlighting the frameshifts in sequences compared with those of the WT. Deletions are shown with dashes and insertions (cytosine) marked in red. The protospacer adjacent motif (PAM) is shown in green bold. The relative percentages of INDEL types was reported in the side brackets.

Preliminary investigation and confirmation of the *SlMYB80* genomic locus sequence in the genetic background of the Microtom cultivar are necessary to support the subsequent design of gRNAs to target complementary regions belonging to functionally predicted domains. Several candidate gRNA binding sites were preselected with the best scores on the basis of the recognition site and the minimum number of predicted off-target effects according to the technical details provided by Concordet and colleagues (Concordet and Haeussler, 2018) through the use of the CRISPOR platform. In particular, specific gRNAs, spanning both coding (CDS) and noncoding (basal promoter) regions, were preselected and chosen for subsequent investigations: the attention was focused on the selection of gRNA_1ex and gRNA_3ex, which are potentially able to target the first and third exons, respectively, of the CDS, and on gRNA_prom around the predicted TSS in the promoter region (**Figure 5A**). The selected gRNAs were synthesized *in vitro*, and their correct assembly *in vitro* with the Cas9 protein was then confirmed through a predicted cleavage observed on the amplified region via the primers Prom_03_Fw/II_ex_01_Rev for gRNA_1ex and the primers III_ex_01_Fw/III_ex_03_Rev for gRNA_3ex and Prom_04_Fw/I_ex_01_Rev for gRNA_prom (for details, see **Supplementary Material**, **Supplementary Table S2**), including the PAM motif sequence and complementary sequence recognized by each gRNA. However, since an *in vitro* validation does not always imply that the complex will also be successfully formed *in vivo*, the next step will be to test the correct recognition and subsequent *in vivo* cleavage.

### 3.6 Targeted deep sequencing to analyse the mutation efficiency of CRISPR RNPs

For each event of transfection, the set quantity of isolated protoplasts was transfected with single RNP complexes preassembled with Cas9, gRNA_1ex, gRNA_3ex, and gRNA_prom, producing the Cas9-gRNA RNP complexes RNP1, RNP3 and RNPP, respectively (for details, see the Materials and Methods section). The transfection procedure was performed at a 1:2 weight ratio of Cas9 and specific gRNA. For transfection negative control samples (NTCs), protoplasts treated with PEG only were used. After 48 h, total genomic DNA was extracted from a pool of protoplasts derived from each transfection event, and to detect *in vivo* the mutation efficiency and patterns at different sites into the *SlMYB80* locus a targeted sequencing-mediated PCR amplification via site-specific primers (i.e., Prom_03_Fw/II_ex_01_Rev for RNP1, III_ex_01_Fw/III_ex_03_Rev for RNP3, and Prom_02_Fw/I_ex_01_Rev for RNPP) was performed in association with TIDE software. Three biological replicates (R1, R2, R3), representing three independent experiments of transfections for each type of RNP complex, were performed, followed by Sanger targeted sequencing. For each biological replicate, the R^2^ value, the total editing efficiency and the type and percentage of most INDELs obtained were also recorded (**Table 2**). Interestingly, the sequence results revealed that various mutation patterns, each with different efficiency rates, were detected only for the RNP1 and RNP3 samples, whereas no mutations were detected with RNPP, with sequences identical to those of the tomato reference genomic sequences and NTC samples tested. In all replicates, the rate of total efficiency for RNP1, which ranged from 15,6 to 24,3%, was higher than that for RNP3 (from 2,8 to 5,6%). Furthermore, the type and relative percentage of the most frequent event (MFE) of INDEL were variable among all the replicates, depending on the distinct targeted sites (**Table 2**). Through Sanger sequencing, it has been possible to investigate the editing-derived genotypes of alleles after transfection with RNP1 and RNP3 in each biological replicate. The WT and the predicted mutated alleles generated, with relative percentage, within exon 1/exon 3 are reported in **Figure 5B** for R1. Similar results for both RNPs are reported in the **Supplementary Material**, **Supplementary Figure S1**, for R2 and R3 biological replicates. Through *in silico* translation software (https://web.expasy.org/translate/), different frameshifts in the *SlMYB80* coding sequence were confirmed (**Figure 5B**), suggesting the possible loss of function of the MYB80 protein.

**Table 2:** Summary of mutations induced in CRISPR/Cas9 RNP-transfected protoplast samples called by TIDE with a p-value<0.001.

Sample name	gRNA type used for transfection	R^2^			Total efficiency (%)			Most Frequent Event (MFE)		
		R1	R2	R3	R1	R2	R3	R1	R2	R3
RNP1	gRNA_1ex	0,86	0,97	0,89	15,6	24,3	17,9	+1(4,7%)	-3(9,5%)	+1(7%)
RNP3	gRNA_3ex	0,95	0,99	0,99	5,6	4,4	2,8	-3(5,6%)	+1(<1%)	-1(1,3%)
RNPP	gRNA_prom	nd	nd	nd	nd	nd	nd	–	–	–
NTC	PEG only	nd	nd	nd	nd	nd	nd	–	–	–

The R^2^ measure of the model fit, the total editing efficiency (%), and the type and relative rate of frequency (%) of INDELs generated are reported for each biologically independent replicate (R1, R2, R3). Nd, Not determined.

### 3.7 Histone signature at gRNA target sites

**Figure 6:**
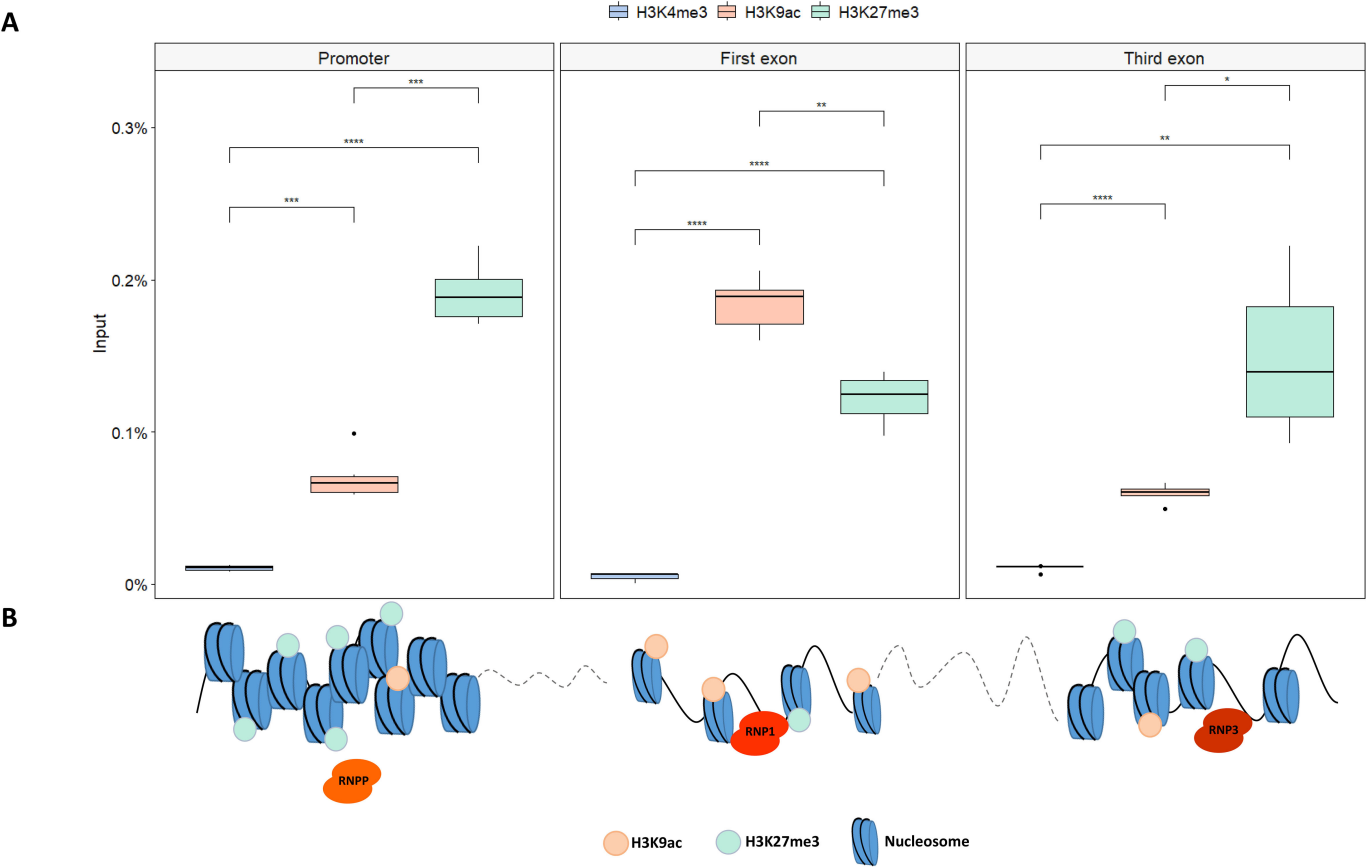
Histone mark distribution in the *SlMYB80* gene. **(A)** Boxplots representing the histone modification analysis at the *SlMYB80* locus by real-time PCR quantification of ChIPed DNA immunoprecipitated with α-H3K4me3, α-H3K9ac, and α-H3K27me3 antibodies on chromatin extracted. The data are reported as percentages of chromatin input, normalized to the background signal (NoAb_measured by omitting the antibody during the ChIP procedure), and three PCR repetitions were performed for each ChIP assay. Asterisks indicate statistically significant changes: *p ≤ 0.05, **p ≤ 0.01, ***p≤ 0.001, ****p≤ 0.0001. **(B)** Schematic representation of the possible predictive model describing the chromatin conformation suggested for three *SlMYB80* locus sites. This suggestion was proposed on the basis of the enrichment levels of each histone mark for H3K9ac and H3K27me3, which determine a conformation suitable or not for the access of each RNP (represented with different orange colour tones) The active mark H3K4me3 has not been reported since it was detected at extremely low values. The proportions of the gene measurements are not to scale.

The distribution of different histone chromatin marks, related to chromatin state, was evaluated along the *SlMYB80* locus through a ChIP assay. Because a correlation between open/closed chromatin conformation and distinct histone modification deposition/distribution is well defined in the literature, the specific enrichments in histone marks correlated with an open conformation (i.e., H3K4me3 and H3K9ac) and a closed conformation (i.e., H3K27me3) were evaluated around the sites in which gRNA_prom, gRNA_1ex and gRNA_3ex were designed. As reported in **Figure 6A**, different enrichments of each histone marks were observed at the investigated sites of the *SlMYB80* locus, suggesting different levels of CRISPR accessibility. By ChIP analysis, a general lack of H3K4me3 marks was detected along the entire locus. A different scenario was observed for the correlated open conformation-related marker H3K9ac, for which an enrichment level was detected at all target sites, with the highest percentage at the first exon and lower and comparable levels at the basal promoter and third exons. In contrast, for the repressive marker H3K27me3, a relatively high level was detected in the promoter region, followed by a decrease in the first and third exon regions. These different enrichment patterns suggest a modulated chromatin conformation along the *SlMYB80* genomic locus, as schematically summarized in **Figure 6B**.

## 4 Discussion

**Figure 7:**
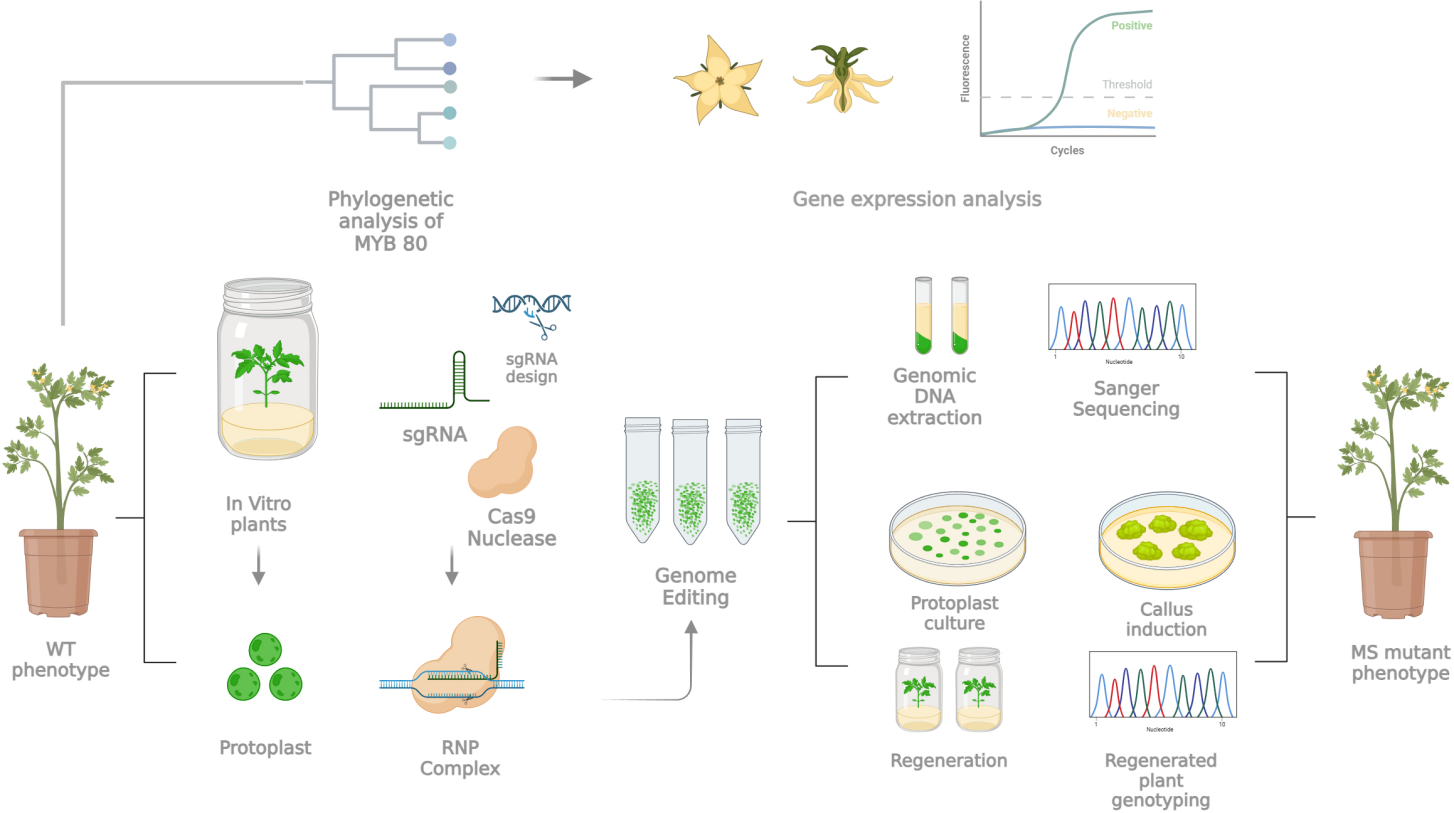
Schematic workflow representing and summarizing the main steps with a key role in the whole experimental pipeline for the generation of tomato DNA-free MS mutants via protoplast-based technology and direct delivery of an RNP complex. For details, see the text.

Over the last decade, GE-based molecular technologies based on the CRISPR/Cas toolbox have contributed greatly to breeding in species with high genome complexity or a long juvenile phase (Pavese et al., 2022). Therefore, it has been possible to perform target mutations to increase key agronomic traits in a shorter time, with the subsequent development of new crop varieties that are more suited to environmental changes or shifting global market demands, as well as their transmission “*from laboratory to field*” (Miladinovic et al., 2021). Among these strong points, there is certainly the opportunity to develop strategies to circumvent transgenic DNA integration, opening a new era in plant precision breeding with transgene-free genome editing technologies and improving not only new molecular tool knowledge but also (agro)biodiversity resources by means of trait engineering. The induction of the male sterility trait still represents a key agronomic strategy (Kim and Zhang, 2018). Tomato is a representative vegetable crop with high economic value in the market because of its high production and consumption worldwide. Furthermore, it may be transformed in an Agrobacterium-dependent way and may serve as a breeding model for horticultural crops because of its short life cycle, small genome and adaptability to *in vitro* cultures (Foolad, 2007). Since the first description (Crane, 1915), MS in tomato has attracted great interest, and approximately fifty spontaneous MS mutants have been investigated (Gorman and McCormick, 1997; Quinet et al., 2014; Sawhney, 2015; Pucci et al., 2017), constituting an excellent system for hybrid seed production. However, the increasing utilization of GE techniques, primarily based on CRISPR/Cas9, has shown the ability to target specific candidate genes to introduce stable mutations. Based on these advancements, we present a case study in which several aspects are evaluated for inducing a site-specific mutagenesis into a candidate gene through a CRISPR/Cas9-mediated toolbox, with the potential to implement male sterility in horticultural crops and generate CRISPR/Cas9-edited DNA-free plants. In particular, since the whole procedure, as schematized in **Figure 7**, results extremely long and is influenced by several technician aspects that could affect the success of the entire process, we decided to discuss the key points of the pipeline for trying to answer the following biological question: while a gene may be considered a strong candidate based on its biological function, does it meet the experimental criteria necessary for successful application?

In this study, we focused on an orthologous gene of the MYB80 transcription factor (*Solyc10g005760*, here defined as *SlMYB80*), which is a promising target for MS in tomato plants, given its potential involvement in the molecular pathways controlling programmed cell death (PCD) in the tapetum (Jung et al., 2020). This biological event has been described in detail in previous studies in both Arabidopsis and rice, highlighting the two molecular pathways responsible for pollen and tapetum development (Fu et al., 2014; Jeong et al., 2014; Zhu et al., 2015; Mishra et al., 2018). To date, an analogous model has been proposed in tomato, describing the involvement and relationships of the main gene actors, some of which belong to the bHLH transcription factor family, and the success of targeted mutagenesis by CRISPR/Cas has also been reported, with direct repercussions on the male sterility of plants (Jung et al., 2020). For these reasons, we evaluated the possibility of applying a similar strategy to this MYB transcription factor member of the pathway to potentially implement the number of future successful of MS tomato plants (Priyadarsini et al., 2025). The first indication which suggests the potential of *SlMYB80* as a good candidate for inducing male sterility in tomato derives via multiple peptide alignment, which highlights a possible relationship, with a high level of peptide conservation, between SlMYB80 and other MYB proteins previously studied and associated with male sterility in other plant families (Zhang et al., 2007; Xu et al., 2014; Palumbo et al., 2019; Pan et al., 2020). As shown in **Figure 1**, the conservation between the main functional R2R3 DNA-binding domains, the 44 amino acids downstream of the R3 region and the variable C-terminal region is confirmed. Such conservation within the MYB protein family is critical for gene functionality, as evidenced by the addition of an EAR motif in the 44 amino acid region, which affects DNA binding or protein−protein interactions, leading to dysfunction of the protein itself (Phan et al., 2012; Xu et al., 2014). As demonstrated for other homologous MYB family genes, the editing of coding sequences, as well as regulatory regions, could serve as an attractive strategy to prevent the synthesis of a functional protein at the pre- or post-transcriptional level (Phan et al., 2011). Furthermore, the level of conservation observed among the five investigated peptides is consistent with the preservation of gene function reported in other species by (Xu et al., 2014).

The second suggestion, lies in its spatial and temporal occurrence of transcripts. The expression pattern analysis revealed that the *SlMYB80* transcript level was greatest in S9 to S11 (**Figure 3B**). According to the literature, during the S9 growth phase, a callose-covered tetrad is formed, whereas in S10, callose is hydrolysed by β-1,3-glucanase, which is secreted by tapetum cells, resulting in the release of free microspores from the tetrad (Brukhin et al., 2003; Pan et al., 2021). For the three genes belonging to the anther developmental cascade described in tomato (*MS10 -Solyc02g079810*- a homologue of *AtDYT1, Ms32 -Solyc01g081100-* a homologue of *AtbHLH91*, and *SlMS -Solyc08g062780-*, a homologue of *AtAMS-like)*, high and specific level of transcription has been established in tapetum cells, especially after meiosis, playing a key role in the development of PCD in tomato (Jeong et al., 2014; Jung et al., 2020; Bao et al., 2022). A similar expression pattern was also observed for *SlMYB80*, with specific tissue co-localization at the level of tapetum cells (**Figure 3C**), supporting the general hypothesis of its potential role during microsporogenesis, which starts with the beginning of the meiosis of the diploid mother cells and ends with the formation of haploid microspores.

In addition to these preliminary evaluations related mainly to more biological role, *SlMYB80* appears to be a good candidate for a CRISPR/Cas-based approach since, along the *SlMYB80* genomic locus, potential gRNAs targeting functional regions (i.e., CDS peptide domains and promoter basal region) are recognized through the identification of PAM motifs and then verified through an *in vitro* assay, confirming the possibility of designing complementary sequences of gRNAs. According to the literature, this phase of investigation is necessary for target site mutagenesis because the possibility of designing several gRNAs that target functional regions, and are capable of correctly assembling the following RNPs, allows us to outline different purposes specific for each case of study. As previously discussed, if, on the one hand, it is understandable that CDS editing induces a frameshift and/or introduces a pre-stop codon, resulting in a truncated protein that is no longer functional, on the other hand, CRISPR−Cas-based promoter editing could be considered a fine-tuning regulation of Sl*MYB80* gene expression in tomato. To date, exploring a strategy of plant promoter editing appears to be a successful approach for studying gene functional diversity and creating new germplasm resources with polymorphic quantitative traits and even entirely new traits based on the same loci via the fine regulation of gene expression. In this context, tomato germplasms with quantitative variations in fruit size, through the regulation of the *SlCLV3* gene and plant structure, which mediate the regulation of the *SlWUS* gene, have also been created (Wang et al., 2021). Notably, a promoter editing strategy was utilized by researchers to create a series of tomato germplasms with different expression patterns of the *SlWOX9* gene, revealing new mechanisms for the divergence of homologous gene function in species evolution (Hendelman et al., 2021).

With the future purpose of obtaining CRISPR/Cas-edited DNA-free plants carrying the MS genetic trait, in addition to a deep investigation of the sequence properties of the potential target gene, we focused on other fundamental aspects for the success of the whole pipeline, which should be carefully optimized, and we report an example of DNA-free transfection in tomato protoplasts by directly delivering RNP complexes. According to the literature (Bortesi and Fischer, 2015), protoplasts are valuable biological models for *in vivo* validation of RNP systems, as they are cells without a cell wall but retain much of the plant’s genetic information. Currently, few DNA-free genome editing methods have been established for tomato, which represents a significant limitation when this important technology is applied for breeding or research purposes. This gap in the literature may be attributed to the fact that a DNA-free genome editing method requires an efficient and reproducible method for protoplast isolation, since the yield and quality of isolated protoplasts have a direct impact on the efficiency rate of RNP delivery, as well as a direct influence on shoot regeneration from single cells, which is still a highly genotype-dependent challenge (Peres et al., 2001). However, promising advances have recently been made in the establishment of a DNA−free genome editing and protoplast regeneration method for cultivated tomato within a few months (Liu et al., 2022) and for wild tomato (*Solanum peruvianum*), which is the closest so far to cultivated tomato (Lin et al., 2022). In our study, we analysed the key variables influencing success in each critical step (e.g., protoplast viability, yield, and efficient transfection), including the concentration of cell wall digestion enzymes, buffer conditions, the osmotic status of protoplasts, the incubation period, and the type of explants used for protoplast isolation, all of which have been methodically investigated, updated and optimized. Specifically, based on a previously published protocol for cultivated tomato protoplast genome editing via RNP-based CRISPR/Cas9 (Nicolia et al., 2021), we implemented the process on the basis of explant age, enzyme digestion temperature and time on our *in vitro*-grown tomato plants under investigation, achieving good and comparable results in terms of protoplast yield and viability, as well as PEG-mediated transfection conditions for the transient expression of the CRISPR/Cas9 system. With the adopted conditions (summarized in **Figure 4**), we successfully increased the yield of protoplasts to 2.6 × 10^6^ per gram of leaf material, which was comparable to previous results obtained for tomato (Morgan and Cocking, 1982; Niedz et al., 1985; Tan et al., 1987a). Furthermore, in agreement with past observations (Tan et al., 1987b), we found that preconditioning of donor plants at 4°C six hours prior to protoplast isolation significantly enhanced protoplast yield and viability by increasing protoplast stability. In contrast to the findings reported in literature (Liu et al., 2022), who suggested that cold treatment had no positive effect, our study demonstrated that this preconditioning treatment was critical for achieving optimal protoplast yield and quality. In fact, in our case, treating the plants at 25°C for 16 hours in the dark did not result in satisfactory protoplasts, as they were relatively weak, broken, and unstable. Regarding the PEG concentration during the transfection phase, a key point of the whole procedure, different patterns among species have been reported in the literature, where reports in which up to 40% PEG solution is employed testify to a higher rate and efficiency of the transfection process. Although good transfection efficiency was observed with 25% PEG solution (Liu et al., 2022), our best results were obtained with a relatively high percentage in PEG solution (40%), suggesting that the relatively high quantity of protoplasts employed during our trials of transfection (2.0 × 10^5^) needs a new optimum of PEG solution, as suggested and reported in other RNP independent transfection experiments in other crops or other plant species (Pavese et al., 2022; Salvagnin et al., 2023).

Establishing a solid starting point regarding transfection efficiency is essential and necessary because a correlation between CRISPR/Cas9-related mutation efficiency and the transformation technique employed has been demonstrated. As previously mentioned, we selected and tested three specific gRNAs able to assemble the RNPP, RNP1 and RNP3 complexes and target three important functional locus sites: the predicted TSS in the basal promoter region, the first and third exons encoding a part of the R2R3 DNA-binding domain, and 44 aa-conserved domains, respectively. To facilitate the highest site-specific mutation frequency in tomato protoplasts, we titrated several ratios (w/w) of Cas9:gRNAs (i.e., 2:1, 1:1, and 1:2), establishing a 1:2 ratio as the optimal ratio for the experiments, resulting in the highest mutation frequency. With this approach, we demonstrated a critical advantage over plasmid-mediated genome editing delivery by titrating the Cas9:gRNA ratio to achieve the maximum mutation frequency (Kanchiswamy et al., 2016). The three corresponding RNP complexes were tested *in vivo* by independently transfecting the same quantity of protoplasts. In detail, distinct sites of *SlMYB80* tend to be targeted with different efficiency resulting in different INDEL mutagenesis efficiencies depending on the target site coordinates. Within the *SlMYB80* locus, the highest editing rates were measured for the first exon (15.6–24.3%) after transfection with RNP1, followed by a lower rate at the third exon (2.8–5.6%) with RNP3 and an undetectable level at the basal promoter utilizing RNPP. The percentage found via RNP1 is comparable to that reported by other studies using single RNP complexes in tomato (9.1–19%), Arabidopsis (16%) and rice (8.4–19%) and higher than that reported in grapevine (0.1%) and apple (0.5–6.9%) (Woo et al., 2015; Malnoy et al., 2016; Liu et al., 2022). According to the literature, a gRNA with a mutation rate higher than 10% in protoplasts could be considered a suitable and promising candidate for the recovery of future edited plants (Brandt et al., 2020). Furthermore, the most frequent events reported in **Figure 5** were the insertion or deletion of single or triplet nucleotides. In any case, these predicted mutation events were related to an alteration of native coding sequence with the possible synthesis of aberrant or truncated nonfunctional polypeptides. The patterns observed in our data are consistent with those reported in the literature, where the most common mutations detected in transfected protoplasts were single nucleotide insertions, followed by deletions of one or three nucleotides (Bortesi et al., 2016).

To gain more insight and provide a molecular explanation for these differences in efficiency between the RNPs employed, we investigated the role of chromatin organization at these three target sites. Numerous studies have highlighted the role of the eukaryotic genome organization machinery as a factor in genome editing outcomes (Jain et al., 2024).

Among the potential biophysical elements, we focused on the influence of the chromatin state and investigated post-translational histone modifications (PTHMs), which are responsible for the fine regulation of more or less open/closed chromatin structural conformations (Jain et al., 2024). Increasing evidence has revealed negative associations between mutagenesis rates induced by the CRISPR/Cas9 system and heterochromatic signatures or low chromatin accessibility in multiple systems, such as yeast (*Saccharomyces cerevisiae*), zebrafish (*Danio rerio*), mouse (*Mus musculus*), human (*Homo sapiens*), and rice (*Oryza sativa*) (Weiss et al., 2022). In our study, we measured the enrichment levels of three histone marks, H3K4me3, H3K9ac and H3K27me3, which were chosen because the literature reports that different chemical modifications (i.e., methylation or acetylation) on different histone lysine residues (i.e., K9, K4 and K27) on histone protein H3 are responsible for chromatin state modulation, defining a specific ‘histone code’ for a gene locus (Liu et al., 2014). On the basis of our results, a specific histone code pattern for *SlMYB80* may be defined: as summarized in **Figure 6B**, a higher level of chromatin condensation was expected at the level of the TSS, with a predominant enrichment of H3K27me3, followed by a conformation that was more open at the level of the first exon, characterized by a decrease in H3K27me3 and a concomitant increase in H3K9ac. This pattern could agree with the different efficiencies observed for RNPP, for which no editing was recorded, and for RNP1, for which an edit was instead recorded. As reported in the literature, histone acetylation acts as a ‘switch’ in the regulation of chromatin conformation via the interconversion of permissive and repressive chromatin structures (Eberharter and Becker, 2002). In our biological example, this point of interconversion could be established around the first exon, allowing the best accessibility to RNP1. This direct correspondence between chromatin conformation and accessibility to the RNP could explain the lower efficiency of RNP3 in targeting a region in which a reduction in H3K9ac and a lower enrichment of H3K27me3 were detected than in the first exon. Interestingly, a slight enrichment in H3K4me3 was detected at all target sites, although H3K4me3 marks regions that are less condensed (i.e., euchromatin regions), like H3K9ac (Wang et al., 2022). We suggest a possible relationship between this apparently controversial pattern and the expression levels of *SlMYB80*: our preliminary investigations confirmed an anther-specific expression pattern, with a nondetectable level in leaf tissue, the same tissue that has been used for isolation and transfection of protoplasts. Several reports indicate that H3K4me3 is correlated with Pol II transcription activation and promotes efficient elongation (Ding et al., 2012; Fromm and Avramova, 2014; Wang et al., 2022). The absence of *SlMYB80* transcription could be the reason for the lack of H3K4me3 (or *vice versa*). This conformation status, in addition to a high level of H3K27me3, which marks facultative heterochromatin regions, makes the target site for RNPP inaccessible *in vivo*. It will be fascinating to further analyse the influence of different chromatin characteristics on CRISPR/Cas9 mutagenesis in future research employing genetic mutants that produce various states of histone modifications. More accurate and effective genome engineering techniques will be possible with a deeper comprehension of the interaction between chromatin dynamics and CRISPR/Cas9.

## 5 Conclusions

Site-specific mutagenesis via CRISPR/Cas-based technology represents a valid and alternative approach to generate targeted genetic variations in crops, introducing new plant traits while ensuring high varietal purity. Several molecular strategies, with the same intent, have been employed and described in the literature to edit target genes involved in male sterility induction and generate edited plants. Furthermore, to date, the idea of obtaining DNA-free genetically edited plants via direct delivery of the RNP complex through protoplast technology represents the most promising approach for avoiding the integration of exogenous DNA (GMO-free). In fact, plant protoplasts constitute a versatile system for CRISPR/Cas9 genome editing, functional analysis of traits, and studies of multiple signalling cascades in several crops. To address our initial biological question—whether a gene considered a good candidate for its proposed biological function can also serve from an experimental standpoint—we focused on *SlMYB80* in tomato. Due to its identification as a potential member of anther development pathways in tomato itself, *SlMYB80* may be considered a theoretical candidate for GE targeting and MS induction.

Our results support this biological involvement, highlighted primarily by a high level of amino acid similarity with other homologues characterized and related to male sterility in other plant species and by spatial/temporal expression of its transcript at the level of tapetum cells. Furthermore, based on the sequence of the genomic locus, we identified three gRNAs able to target three fundamental functional sites, including the predicted TSS, the region encoding R2R3 and the 44-aa conserved domains. All these investigated gRNAs were able to assemble *in vitro* with the Cas9 protein, generate the respective functional RNP complexes, and efficiently recognize the specific target sequences. To evaluate these methods under *in vivo* conditions and provide an opportunity to develop DNA-free genome-edited crop plants from single protoplast cells, an efficient protoplast isolation and transfection protocol via a PEG-mediated transfection system was established and described in the tomato cultivar Microtom. For each CRISPR RNP under investigation, we relieved different levels of mutagenesis efficiency at different sites into the same locus, suggesting an additional level of ‘control’. Since the mutation efficiency was found to vary in relation to the different enrichment levels of histone marks distributed along the gene locus, our observations suggest that the nonsequence features influence CRISPR/Cas9 mutagenesis for SlMYB80, and for this reason, we believe that this epigenetic-related aspect is a key point of evaluation of the predictability of the chosen toolbox so that it can be considered an effective strategy. On the basis of these prediction starting points, further studies are now needed to optimize the plant regeneration phase from CRISPR RNPs to transform protoplasts to explore the applications of this technology at the field level.

## Data Availability

The original contributions presented in the study are included in the article/**Supplementary Material**. Further inquiries can be directed to the corresponding author/s.
