# Supplementary material for: Unlocking male sterility in horticultural crops through gene editing technology for precision breeding applications: presentation of a case study in tomato
Source: Front Plant Sci. 2025 Mar 6;16:1549136. doi: 10.3389/fpls.2025.1549136 (PMC11924944; doi:10.3389/fpls.2025.1549136)
Supplement: Supplementary file 1 [file DataSheet1.docx]

**Protocol S1:** Detail description of protocol for isolation and transfection of protoplasts. Chemical compositions of solutions employed are also reported below.

**Day 0: Pre -Treatments for plant conditioning**

- 24 hours before the start of the experiment, cover the selected plants in aluminum foil and place them in a controlled environment chamber at 24 degrees.
- Transfer the plants at 4°C at least 8 hours before starting the experiment.

**Day 1: Tissue Preparation and Enzymatic Digestion**

- Under sterile conditions, take around 20-30 tomato young leaves, weight them in a petri dish and submerge them in the enzymatic extraction buffer at a ratio of 5 mL per 150 mg of leaf tissue.
- Cut them finely into strips using sterilized blades.
- Incubate the solution in darkness at room temperature with gentle agitation for 16-18 hours. The incubation period allows the enzymatic digestion of the leaf tissues, indicated by a change in solution color to green.

**Day 2: Protoplast Collection and Purification**

- Next morning, add an equal volume of W5 solution to the green solution.
- Filter the resulting solution using sterile 100 μM Nylon mesh, is advisable made the carefully transfer of the protoplast suspension onto the filter using a Pasteur pipette.
- Gently transfer the filtered protoplasts to 15 mL Falcon tubes.
- Set the centrifuge parameters to 100 g for 5 minutes, with an acceleration setting of 2 and a deceleration setting of 0. Discard the liquid, and resuspend the pelleted protoplasts in 5 mL of W5 solution.
- Once resuspended, carefully overlay the protoplast suspension onto a CPW + 21% saccharose solution, taking care to not mix the two different phases, centrifuge them at 100Xg for 5 minutes
- Extract the new formed green layer containing the protoplasts and transfer to new Falcon tubes.
- Wash the protoplasts by resuspending them in 10 mL of W5 solution, followed by centrifugation at 100Xg for 5 minutes.
- Repeat the washing step one more time, after the last one resuspend the protoplasts in 1 ml of MMG solution.
- Count the isolated protoplasts using a hemocytometer to determine the yield.
- After it, the resulted protoplasts must be stained with 0.01% fluorescein diacetate (FDA) for five minutes in order to measure the viability of them.

**RNP Complex Assembly steps:**

1. **sgRNA DNA Template Preparation**
   Combine 12.5 μL of 2× Phusion High-Fidelity PCR Master Mix, 1 μL of Tracr Fragment + T7 Primer Mix, 1 μL of 0.3 μM Target (F-R) primer mix, and 10.5 μL of nuclease-free water. Subject the mixture to thermocycling conditions: 10 seconds at 98 °C, followed by 32 cycles of 5 seconds at 98 °C and 15 seconds at 55 °C, and a final extension of 1 minute at 72 °C. Verify successful assembly by running a 5 μL aliquot on a 2% agarose gel, ensuring a 120 bp band is visible. Purify the remaining sgRNA DNA using a PCR purification kit and measure the DNA concentration with a micro-volume spectrophotometer.
2. **In Vitro Transcription**
   Prepare the transcription reaction by mixing 8 μL of NTP mix, 40 ng of sgRNA DNA template, 4 μL of 5× TranscriptAid reaction buffer, 2 μL of TranscriptAid enzyme mix, and RNase-free water to a final volume of 20 μL. Incubate the reaction at 37 °C for 4 hours, then add 1 U of DNase I and continue incubation at 37 °C for an additional 15 minutes. Purify the sgRNA using the GeneArt Cleanup Kit according to the manufacturer’s instructions.
3. **Quality Control**
   Evaluate the quality of the sgRNA by performing gel electrophoresis. Prepare a dilution of the sgRNA sample, add loading dye, and heat at 70 °C for 10 minutes to prevent secondary structure formation. Load 2 μL of the sample onto a 2% E-Gel EX agarose gel and run the gel, confirming a 100 bp band. Measure the sgRNA concentration with a Qubit fluorometer and store at -80 °C until required.
4. **RNP Complex Assembly**
   To assembly the RNP complex, 30 μg of sgRNA with 15 μg of TrueCut Cas9 Protein v2 must be combined in a total volume of 20 μL. Gently mix by pipetting and incubate at room temperature for 10 minutes to facilitate the formation of the RNP complex.
5. **Protoplast Transfection**
   To initiate transfection, prepare a suspension of 250,000 protoplasts.

- Carefully add the pre-formed RNP complex at a molar ratio of 1:2 (Cas9: sgRNA) to the protoplast suspension, ensuring thorough but gentle mixing to avoid damaging the protoplasts.
- Subsequently, introduce 220 μL of a 40% PEG-Ca²⁺ solution, and incubate the mixture at room temperature for 10 minutes.
- After incubation, add gently 880 μL of W5 solution, centrifuge the mixture at 100 g for 5 minutes, and carefully aspirate the supernatant. Suspend the resultant pellet with 2 mL of W5 solution, followed by a second centrifugation under identical conditions.
- After removing the supernatant, suspend the protoplast pellet in 1 mL of W1 solution.
- Genomic DNA can be extracted from the transfected protoplasts after three days.

| \| **EXTRACTION BUFFER** \| \| \| --- \| --- \| \| **ELEMENT** \| **CONCENTRATION** \| \| MACEROENZYME \| 0.50% \| \| CELLULASE \| 1% \| \| PECTOLIASE \| 0.05% \| \| BSA \| 0.1% \| \| D-MANNITOL \| 0.4 M \| \| MES \| 20 mM \| \| CaCl_2_ \| 10 mM \| \| KCl \| 20 mM \| \| Water \| - \|   **BUFFER W5 ^a^** | |
| --- | --- | --- | --- | --- | --- | --- | --- | --- | --- | --- | --- | --- | --- | --- | --- | --- | --- | --- | --- | --- | --- | --- | --- |
| **ELEMENT** | **CONCENTRATION** |
| NaCl | 154 mM |
| MES | 2 mM |
| CaCl_2_ | 125 mM |
| KCl | 5 mM |
| Glucose | 5 mM |
| Water | - |

**^a^Revised from Cheng & Nakata, 2020**

| **CPW ^b^** | |
| --- | --- |
| **ELEMENT** | **CONCENTRATION** |
| CaCl_2_ | 148 mg/L |
| CuSO_4_ * 5H_2_O | 0.025 mg/L |
| MgSO_4_ * 7 H_2_O | 246 mg/L |
| MES | 976 mg/L |
| KNO_3_ | 101 mg/L |
| KI | 0.160 mg/L |
| KH_2_PO_4_ | 27.2 mg/L |
| D-MANNITOL | 130 mg/L |
| Saccharose | 21% |
| Water | - |
| **^b^Revised from Sangra et al., 2019**  **MMG ^c^** | |
| **ELEMENT** | **CONCENTRATION** |
| D-Mannitol | 500 mM |
| MES | 4 mM |
| MgCl_2_ | 15 mM |
| Water | - |

**^c^Revised from Yoo et al., 2007**

| **W1** | |
| --- | --- |
| **ELEMENT** | **CONCENTRATION** |
| D-Mannitol | 500 mM |
| MES | 4 mM |
| KCl | 20 mM |
| Water | - |

| **PEG 4000** | |
| --- | --- |
| **ELEMENT** | **CONCENTRATION** |
| D-Mannitol | 200 mM |
| CaCl_2_ | 100 mM |
| PEG 4000 | 40% |
| Water | - |

**References:**

Cheng N and Nakata PA (2020) Development of a rapid and efficient protoplast isolation and transfection method for chickpea (*Cicer arietinum*). *MethodsX*. 8;7:101025.

<https://doi:10.1016/j.mex.2020.101025>

Sangra, A, Shahin, L and Dhir, SK (2019) Optimization of Isolation and Culture of Protoplasts in Alfalfa

(Medicago sativa) Cultivar Regen-SY. *American Journal of Plant Sciences*, 10, 1206-1219.

<https://doi.org/10.4236/ajps.2019.107086>

Yoo, SD, Cho, YH and Sheen, J (2007) Arabidopsis mesophyll protoplasts: a versatile cell system for transient gene expression analysis. *Nature Protocols,* 2, 1565–1572.

<https://doi.org/10.1038/nprot.2007.199>
