# Supplementary figures and images for: Unlocking male sterility in horticultural crops through gene editing technology for precision breeding applications: presentation of a case study in tomato
Source: Front Plant Sci. 2025 Mar 6;16:1549136. doi: 10.3389/fpls.2025.1549136 (PMC11924944; doi:10.3389/fpls.2025.1549136)

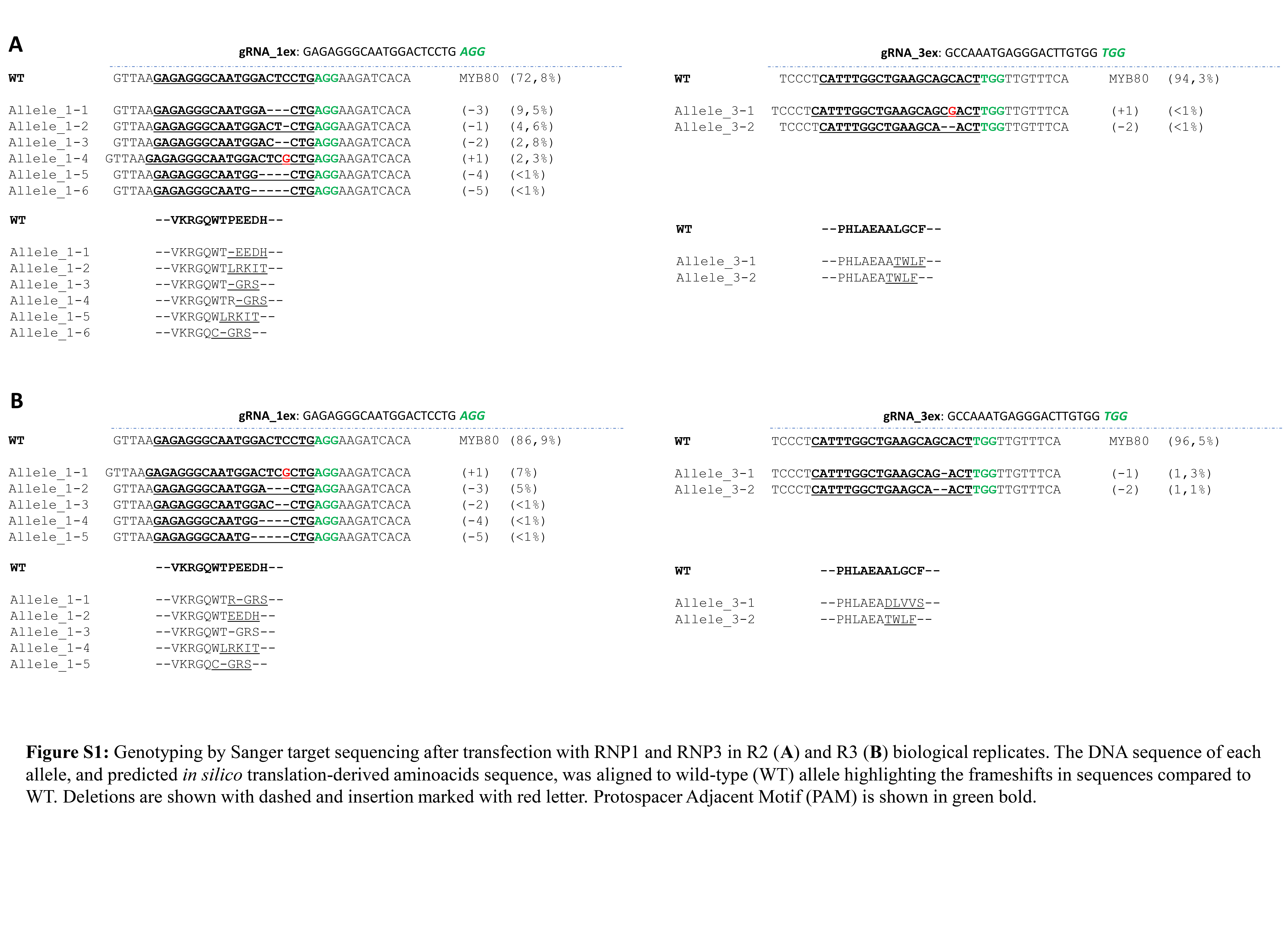

Supplement: Supplementary file 2 [file Image1.jpeg]
