# Supplementary material for: Unlocking male sterility in horticultural crops through gene editing technology for precision breeding applications: presentation of a case study in tomato
Source: Front Plant Sci. 2025 Mar 6;16:1549136. doi: 10.3389/fpls.2025.1549136 (PMC11924944; doi:10.3389/fpls.2025.1549136)
Supplement: Supplementary file 3 [file Table1.docx]

**Table S1:** Primer list and relative informations. ^a^Locus position: genomic position relatives to the predicted ATG (+1).

| **Gene Locus** | **Primer name** | **Primer sequence 5'>3'** | **^a^Locus position** |
| --- | --- | --- | --- |
| glyceraldehyde-3-phosphate dehydrogenase  Actin  Tubulin | GADPH_Fw | CTGCTCTCAGTAGCCAACAC | see Lodval and Lillo, 2009 |
|  | GADPH_Rev | CTTCCTCCAATAGCAAGAGGTTT |  |
|  | Act Fw | GAATAGCATAAGATGGCAGACG |  |
|  | Act Rev | ATACCCACCATCACACCAGTTAT |  |
|  | Tub Fw | AACCTCCATTCAGGAGATGTTT |  |
|  | Tub Rev | TCTGCTGTAGCATCCTGGTATT |  |
| SlMYB80 (Solyc10g005760) | Prom_01_Fw | GGAACTATATTGTACCACTCATGCA | -1159 |
|  | Prom_02_Fw | CATGTTAGCGATGAAAGAAATTAAAAGAACTCG | -847 |
|  | Prom_03_Fw | GTCATGCACCTTTGATGCTCAC | -178 |
|  | Prom_04_Fw | CCCTTAGCTTCGCCCTGTTAAGTGTTG | -734 |
|  | Prom_05_Rev | GTGAGCATCAAAGGTGCATGAC | -156 |
|  | I_ex_01_Fw | ATGGGAAGAATTCCATGTTGTGAAAAGGAC | +1 |
|  | I_ex_01_Rev | GTCCTTTTCACAACATGGAATTCTTCCCAT | +30 |
|  | II_ex_01_Rev | GGCCATGTTTGAGATCAGGCCTAAGGT | +575 |
|  | III_ex_01_Fw | TGTGATAGCTGCTCAACTTCCCGGT | +1153 |
|  | III_ex_02_Rev | TGAAACAACCAAGTGCTGCTTCAGC | +1353 |
|  | III_ex_03_Rev | TCAAACCATTGGATTCATTAGATCATCAGATGATATG | +1895 |

Note: To evaluate the performance of potential housekeeping primers (GAPDH, Act, Tub), one microgram of total RNAs, extracted from each biological sample (whole buds at different growth stages, isolated anthers and young leaves), was reverse-transcribed using PrimeScript™ RT Reagent Kit following the manufacturer’s protocol. After a serial dilution, 1 µl of cDNA was then utilized for following semi quantitative RT-PCR reactions, using the Platinum Multiplex PCR Master Mix (Applied Biosystems, Carlsbad, CA, United States) with 0.1 μM of each forward and reverse specific primers. The PCR assay were performed as follows: 1 cycle at 95°C for 1 min, 25 cycles at 95°C for 15 s, 55°C for 30 s, 72°C for (1 kb/ min), and 1 cycle at 72°C for 5 min. PCR assays were performed in a total volume of 20 μl and the amplification signal was visualized on 2% agarose gel.
