# Supplementary material for: Unlocking male sterility in horticultural crops through gene editing technology for precision breeding applications: presentation of a case study in tomato
Source: Front Plant Sci. 2025 Mar 6;16:1549136. doi: 10.3389/fpls.2025.1549136 (PMC11924944; doi:10.3389/fpls.2025.1549136)
Supplement: Supplementary file 4 [file Table2.docx]

**Table S2:** gRNA primer list and relative informations. ^a^Locus position: genomic position relatives to the predicted ATG (+1). ^b^Length of preamplified DNA target before *in vitro* cleavage. ^c^Expected length of fragments (pb) due to cleavage. For major details, see section 3.6 of the text.

| gRNA name | Sequence 5'>3' | PAM | ^a^Locus position | ^b^Length of amplified target (bp) | ^c^Length of fragments (bp) |
| --- | --- | --- | --- | --- | --- |
| gRNA_1ex | GAGAGGGCAATGGACTCCTG | AGG | +58 | 753 | 236+517 |
| gRNA_3ex | GCCAAATGAGGGACTTGTGG | TGG | +1330 | 742 | 157+585 |
| gRNA_prom | GTCGAGCACTGAAACACAAC | CGG | -546 | 764 | 208+556 |
